# Sigma-1 Receptor Activation by Fluvoxamine Ameliorates ER Stress, Synaptic Dysfunction and Behavioral Deficits in a Ketamine Model of Schizophrenia

**DOI:** 10.1007/s11481-025-10231-4

**Published:** 2025-07-25

**Authors:** Mariam K. Ahmed, Kareem Abdou, Weam W. Ibrahim, Ahmed F. Mohamed, Noha A. El-Boghdady

**Affiliations:** 1 Department of Biochemistry, Faculty of Pharmacy, Cairo University, Kasr El Aini St, Cairo, 11562 Egypt; 2 Department of Pharmacology and Toxicology, Faculty of Pharmacy, Cairo University, Cairo, Egypt; 3 College of Pharmacy, Al-Ain University, Abu Dhabi, UAE; 4 Faculty of Pharmacy, King Salman International University (KSIU), Ras Sedr, 46612 South Sinai Egypt

**Keywords:** Schizophrenia, NMDAR, ER Stress, Chaperones, Sigma-1 receptor, Misfolded proteins

## Abstract

**Abstract:**

Endoplasmic reticulum (ER) stress and misfolded proteins accumulation are recognized as central factors in the development of psychiatric disorders. This study evaluated the potential therapeutic effect of fluvoxamine, a potent sigma-1 receptor agonist in alleviating protein misfolding and the subsequent ER stress in ketamine–induced model of schizophrenia. NE100 hydrochloride, a sigma-1 receptor blocker, was used to investigate the role of this receptor in fluvoxamine-mediated effects. Rat model of schizophrenia was induced by intraperitoneal administration of ketamine (30 mg/kg/day) for 5 consecutive days. Then, rats were treated with fluvoxamine (30 mg/kg/day, p.o), with or without NE100 (1 mg/kg/day, i.p), for 14 days. Fluvoxamine improved the learning abilities, cognitive flexibility, and sociability functions of ketamine-subjected rats as evidenced in Morris water maze and three-chamber social interaction tests. It mitigated ketamine-induced inhibition of nNOS/PSD-95/NMDAR signaling pathway, thus augmented the function of parvalbumin-GABAergic neurons as indicated by increasing the prefrontal cortical levels of parvalbumin and GAD67. Fluvoxamine also attenuated the prefrontal cortical production of unfolded protein response markers, namely, IRE-1, PERK, and ATF-6, highlighting its ability to alleviate ER stress. Further, it exerted anti-apoptotic and anti-inflammatory effects as shown by lowering Iba-1, tumor necrosis factor-α (TNF-α), Bax, and caspase-12 levels contrary to elevating Bcl-2. Additionally, it attenuated the histopathological alterations in prefrontal cortical neurons. Noteworthy, the co-administration of NE100 reduced the advantageous effects of fluvoxamine, indicating the involvement of sigma-1 receptor in mediating the observed antipsychotic effects. Thus, sigma-1-mediated signaling pathways could be therapeutic targets for preventing or slowing schizophrenia progression.

**Graphical Abstract:**

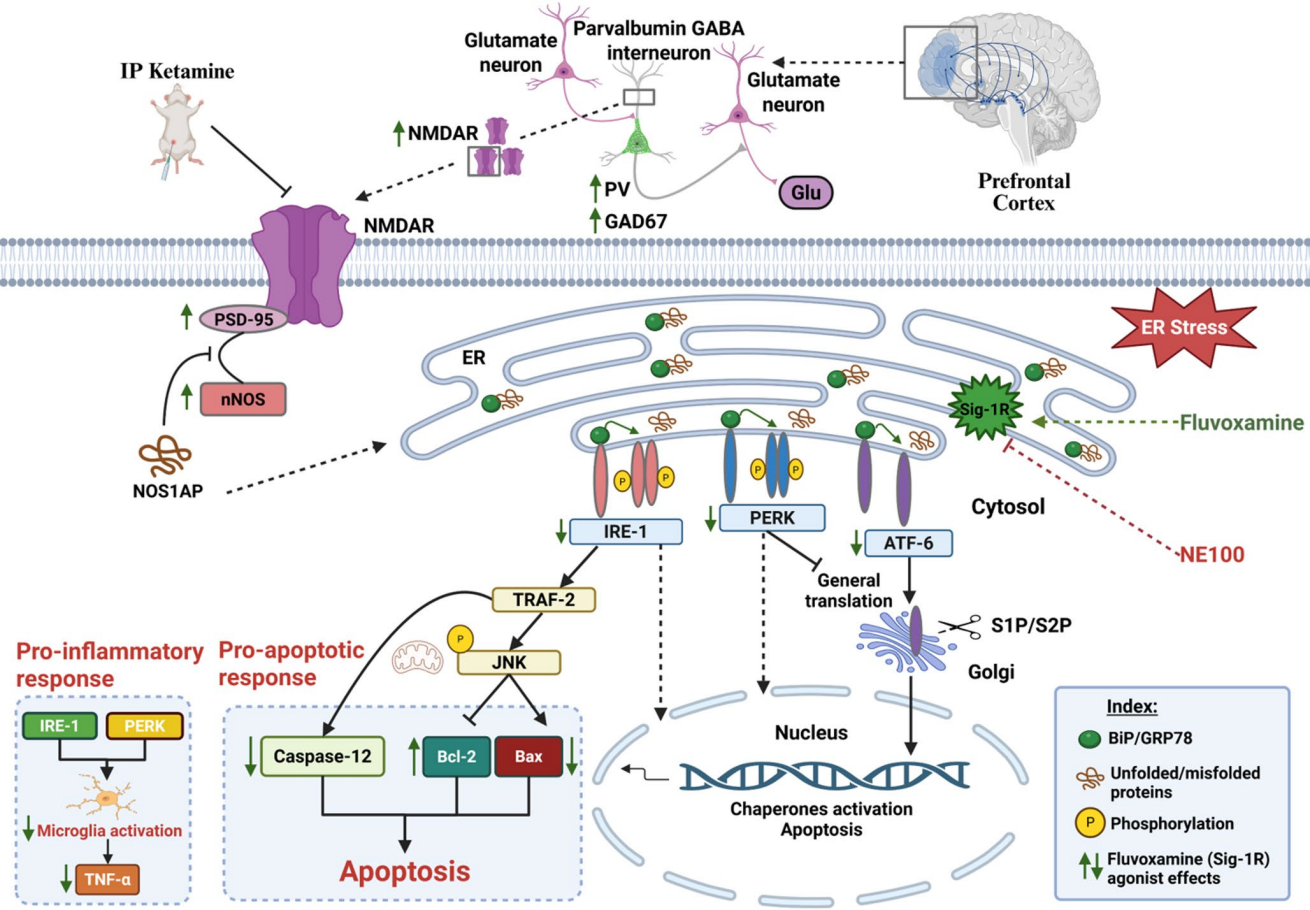

**Supplementary Information:**

The online version contains supplementary material available at 10.1007/s11481-025-10231-4.

## Introduction

Schizophrenia is a chronic and severe neuropsychiatric disorder that disturbs individuals’ behavior and quality of life and affects roughly 1% of the world population (Xie et al. 2020; Shangase et al. 2025). The symptoms of schizophrenia are classified into positive symptoms, negative symptoms, and cognitive symptoms (Farkhakfar et al. 2023). While positive symptoms consist of delusions, hallucinations, and delirium, negative symptoms include anhedonia, affective blunting and social withdrawal (Ibrahim et al. 2022; Samizadeh et al. 2024). Cognitive impairment is considered as one of the core symptoms of schizophrenia and is seen in almost 80% of schizophrenia patients. The main symptoms of cognitive impairment are disorientation, disorganization, defective working memory and lack of attention, which can influence the patients’ daily life activities, social abilities, and work execution (Dong et al. 2021). The etiology of schizophrenia is not fully understood, but numerous theories have been suggested, linking the disease to various genetic and environmental factors (Adamu et al. 2023).

Despite advances in pharmacotherapy, currently used antipsychotics—including second-generation agents (SGAs)—remain largely ineffective in treating the primary negative and cognitive symptoms of schizophrenia (Grinchii and Dremencov 2020; McCutcheon et al. 2023). While some SGAs, such as risperidone and cariprazine, may indirectly improve the secondary negative symptoms through their effects on mood or positive symptoms, their direct impact on core negative or cognitive deficits is limited (Kirkpatrick et al. 2006; Millan et al. 2014; Fusar-Poli et al. 2015). Evidence from large trials and meta-analyses shows that no currently approved antipsychotic significantly enhances cognitive function (Keefe et al. 2007, Millan et al. 2012). Although cariprazine has demonstrated modest efficacy in one randomized trial targeting negative symptoms (Németh et al. 2017), no pharmacological agent has shown consistent, robust effects or received regulatory approval specifically for negative or cognitive symptom domains. This underscores an urgent, unmet clinical need in schizophrenia treatment.

The endoplasmic reticulum (ER) is considered as a large membranous cellular organelle. It can be found in all eukaryotic cells, and it is the main site of protein folding in cells (Schwarz and Blower 2016). Physiologic stresses, such as increased secretory load, or pathological stresses, including the presence of mutated proteins that are not able to fold adequately in the ER resulting in an imbalance between the demand for protein folding and the protein folding capacity of the ER, consequently causing ER stress (Lin et al. 2008). Eukaryotic cells have developed a set of signal transduction pathways collectively referred to as the unfolded protein response (UPR) (Wang and Kaufman 2016). The UPR is controlled by three ER-transmembrane stress sensors, namely inositol-requiring enzyme 1 (IRE-1), protein kinase R-like ER kinase (PERK), and activating transcription factor 6 (ATF-6) (Hetz et al. 2020). IRE-1 is a type I transmembrane protein with a cytosolic serine/threonine kinase domain (Cao and Kaufman 2012). The kinase domain of IRE-1 interacts with the tumor necrosis factor receptor associated factor-2 (TRAF-2) adaptor molecule and activates the apoptosis signal-regulating kinase 1 (ASK1), leading to the phosphorylation and activation of c-Jun N-terminal protein kinase (JNK) (Park et al. 2017). JNK has been shown to induce cell death in response to ER stress (Barr and Bogoyevitch 2001). Similarly, the PERK pathway of the UPR also triggers proapoptotic responses after its activation (Hiramatsu et al. 2015). In response to ER stress, PERK tends to activate a kinase function in its cytosolic domain (Jaud et al. 2020). The only identified target of its kinase activity is the eukaryotic initiation factor 2α (eIF2α) which is a ubiquitous cofactor essential for the assembly of 80 S ribosomes at the initiation codon of mRNAs to initiate protein synthesis (Baird and Wek 2012; Hao et al. 2020). Phosphorylation of eIF2α by kinases such as PERK, inhibits its function dropping protein synthesis in the cell as ribosomes fail to assemble effectively on mRNAs (DuRose et al. 2009). Thus, PERK signaling protects the cell from protein misfolding in the ER by creating a global slowdown of protein translation (Harding et al. 2000). ATF-6 is a type II transmembrane protein containing a cAMP-responsive element-binding protein/ATF basic leucine zipper domain (Wang et al. 2000; Cui et al. 2016). In response to ER stress, ATF-6 is translocated from the ER to Golgi apparatus, where specific proteases cleave the transmembrane domain liberating the cytosolic fragment of ATF-6. This fragment then moves to the nucleus, where it acts as a transcription factor to upregulate UPR target genes. In this way, ATF-6 is thought to protect the cell from ER stress (Sharma et al. 2019). Physiological processes that require a high rate of protein synthesis and secretion must preserve the activation of the UPR’s adaptive programs without triggering cell death pathways. However, above a certain threshold, persistent ER stress causes increased incidence of apoptosis and subsequently leads to the development of pathological diseases including schizophrenia (Tabas and Ron 2011; Almanza et al. 2019).

Molecular chaperones are various families of multidomain proteins that have evolved to assist nascent proteins to reach their native fold. Moreover, they tend to protect protein subunits during the assembly of complexes through preventing protein aggregation or mediating targeted unfolding and disassembly (Saibil 2013). Sigma-1 receptors (Sig-1Rs) are molecular chaperones primarily found in the ER but also present in the plasma membrane. They are highly expressed in the CNS and play a vital role in various cellular functions including cell differentiation, neurogenesis, activation of microglia, and protein quality control. (Tsai et al. 2014). Disturbance in any of the abovementioned cellular processes could possibly accelerate the progression of many neurological disorders including neuropsychiatric diseases such as schizophrenia (Hayashi and Su 2007).

Sub-anesthetic dose of ketamine is a well-established animal model of schizophrenia based on n-methyl-d-aspartate receptor (NMDAR) antagonism. There are several pieces of evidence that support the NMDAR hypofunction theory of schizophrenia is supported including the psychotogenic action of ketamine producing both positive and negative symptoms resembling those associated with schizophrenia (Chatterjee et al. 2012; Lee and Zhou 2019). It was suggested that Sig-1R molecular chaperone has the ability to enhance the function of NMDAR (Pabba et al. 2014; Sałaciak and Pytka 2022).

Fluvoxamine is a selective serotonin reuptake inhibitor (SSRI) widely used in the treatment of depression and other psychiatric disorders (Wilde et al. 1993). It was one of the first used SSRIs and is still used in clinical practice. While fluvoxamine was originally developed to treat depression, its main use now is in managing anxiety disorders, especially obsessive-compulsive disorder (OCD) (Irons 2005). In 1996, it was established that certain SSRIs including fluvoxamine exhibit high-to-moderate affinity for Sig-1Rs in the rat brain (Narita et al. 1996). Moreover, subsequent research utilizing murine models and cellular culture system demonstrated that fluvoxamine act as agonist of Sig-1R (Hashimoto et al. 2007; Nishimura et al. 2008; Ishima et al. 2009). Fluvoxamine has shown to have the strongest effect among all available SSRIs on the molecular chaperones Sig-1Rs with low-nanomolar affinity (Sukhatme et al. 2021). It was reported that Sig-1R agonists could be used for treating neuropsychiatric disorders through tackling factors like oxidative stress, inflammation, disruptions in Ca^2+^ balance, and increased production of normal or misfolded proteins that can result in the buildup of unfolded proteins, and eventually, the onset of neuropsychiatric disorders including schizophrenia (Hashimoto 2015). Fluvoxamine attaches to Sig-1Rs in the ER, leading to separation of the Sig-1R from its binding immunoglobulin protein (BiP) complex. This unbound Sig-1R can activate chaperone activity, leading to neuroprotection (Hayashi and Su 2007).

Although mounting evidence suggests that ER stress and abnormal protein folding are implicated in schizophrenia, the potential of Sig-1R to counteract these effects is not yet fully understood. In particular, while some Sig-1R agonists have shown to modulate NMDA receptors and reduce psychotic symptoms, their role in reversing ER stress-related neurodegeneration in schizophrenia remains uncertain. Accordingly, research is needed to determine whether fluvoxamine’s Sig-1R activation can reduce ER stress-related damage and improve cognitive and social deficits in schizophrenia, potentially offering new treatments for resistant and negative symptoms.

Therefore, the present study was constructed to evaluate the effect of fluvoxamine on ketamine–induced schizophrenia with the emphasis of investigating its role on ER stress sensors. Meanwhile, NE100, a Sig-1R receptor blocker, was used to investigate the role of this receptor on the observed fluvoxamine’s effect.

## Materials and Methods

### Declarations

All experimental and investigational procedures were compliant with the recommendations of the Guide for the Care and Use of Laboratory Animals, published by the US National Institutes of Health (Publication No. 85 − 23, revised 2011), and were approved by the Ethics Committee for Animal Experimentation at the Faculty of Pharmacy, Cairo University (Cairo, Egypt) (permit number: BC-3425). Along the experimental period, every attempt was made to limit the animals’ suffering.

### Animals

The experimental study was carried out using forty-eight male Wistar rats, aged 6–8 weeks and weighed 150–200 g, which were obtained from the animal colony of the National Research Institute (Giza, Egypt). Rats were maintained in an insulated animal house with optimal environmental settings, including constant humidity of 60 ± 10% and temperature of 25 ± 2 °C with 12 h light/dark cycle (lights on at 7:00 a.m.) and they are provided with water and rat chow ad libitum.

### Drugs and Chemicals

Fluvoxamine maleate was provided from Abbott Laboratories, Abbott Park, IL, USA and ketamine hydrochloride (50 mg/ml) was obtained from Rotexmedica, Trittau, Germany. NE100 hydrochloride was provided from Tocris Bioscience, Bristol, United Kingdom (Batch No.: 3B/287114). Fluvoxamine and NE100 hydrochloride were freshly prepared daily using physiological saline. All other chemicals used were of the highest available analytical quality.

### Experimental Design

**Fig. 1:**
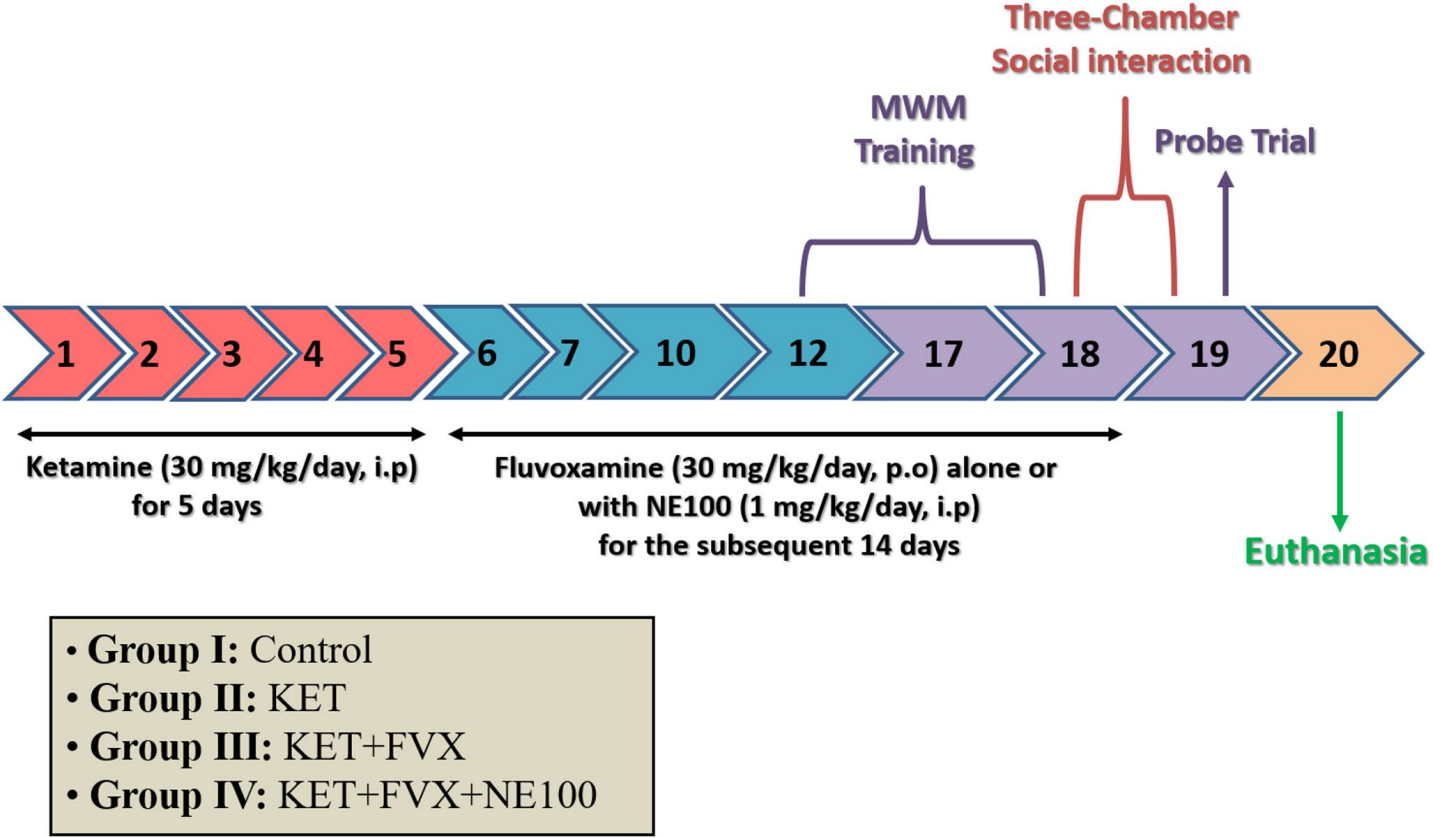
Experimental Design

Rats were accustomed for 1 week in the animal house before initiating the experiment. Then, they were distributed randomly by a technical assistant who was not a part of the study into four groups (*n* = 12/group) as follows: normal group (Control), ketamine model group (KET), ketamine + fluvoxamine group (KET + FVX), and ketamine + fluvoxamine + NE100 group (KET + FVX + NE100), and they were treated as indicated in Fig. 1. The rat model of psychosis was done by the intraperitoneal administration of ketamine subanesthetic dose (30 mg/kg/day) for 5 consecutive days (Estaphan et al. 2021). Then, on the 6th day, rats were orally treated with fluvoxamine at a dose of 30 mg/kg/day (Uslu et al. 2023) for the subsequent 14 days. NE100 (1 mg/kg/day) was given intraperitoneally for 14 days prior to fluvoxamine with almost 15 min (Narita et al. 2001). Fluvoxamine is prepared in concentration of 3 mg/ml in normal saline and administered as 1 ml/100 gm body weight of rat. Three days before the end of experiment, rats were behaviorally tested using three-chamber social interaction test (18th and 19th day) and Morris water maze (MWM) test (19th day). All tests were conducted during the animal’s light cycle, in the previously mentioned sequence from the least stressful test to the most stressful one with a 2-h rest period between the tests on days with overlapped tests.

### Behavioral Tests

#### Three-chamber Social Interaction Test

Social interaction impairment is a unique characteristic of animals suffering from schizophrenia-like disorder. On the experiment day, animals were socially isolated in plastic cages with dimensions (43 × 28 × 15 cm) for 3 h before the experiment. The test was performed in three connected rectangular chambers; each compartment has dimensions of 23 × 40 × 22 cm each. An access to each compartment was provided by two slits measured 9 cm^2^ each. The central compartment was the starting point for each test. In the center of each side compartment, a 10 cm diameter wire cage was placed. The subject rat was allowed to explore the three chambers for 5 min as a “habituation period”.

Then, the sociability test started by putting the stranger rat into one of the two wire cages, this rat represented “novel rat 1”, while leaving the other wire cage which represented the “novel object” empty. The subject rat was then allowed to roam around the three chambers for 10 min. The time of interaction with the “novel rat 1” or the “novel object” in the sociability test was measured and used to calculate the sociability index (SI) as follows:

$$\:SI=\frac{\text{T}\text{i}\text{m}\text{e}\:\text{e}\text{x}\text{p}\text{l}\text{o}\text{r}\text{i}\text{n}\text{g}\:\text{n}\text{o}\text{v}\text{e}\text{l}\:\text{r}\text{a}\text{t}\:1\:-\:\text{t}\text{i}\text{m}\text{e}\:\text{e}\text{x}\text{p}\text{l}\text{o}\text{r}\text{i}\text{n}\text{g}\:\text{n}\text{o}\text{v}\text{e}\text{l}\:\text{o}\text{b}\text{j}\text{e}\text{c}\text{t}}{\text{T}\text{i}\text{m}\text{e}\:\text{e}\text{x}\text{p}\text{l}\text{o}\text{r}\text{i}\text{n}\text{g}\:\text{n}\text{o}\text{v}\text{e}\text{l}\:\text{r}\text{a}\text{t}\:1\:+\:\text{t}\text{i}\text{m}\text{e}\:\text{e}\text{x}\text{p}\text{l}\text{o}\text{r}\text{i}\text{n}\text{g}\:\text{n}\text{o}\text{v}\text{e}\text{l}\:\text{o}\text{b}\text{j}\text{e}\text{c}\text{t}}$$

Afterwards, the social novelty preference test is immediately started for an additional 10 min, where a new unfamiliar rat was added to the previously empty cage and is now considered the “novel rat 2”, whereas the previous stranger rat “novel rat 1” became the “familiar rat” and remains in its place. The time spent exploring the “novel rat 2” or the “familiar rat” in the social novelty preference test was utilized to calculate the social novelty preference index (SNI) as follows (Bambini-Junior et al. 2014):

$$\:SNI=\frac{\text{T}\text{i}\text{m}\text{e}\:\text{e}\text{x}\text{p}\text{l}\text{o}\text{r}\text{i}\text{n}\text{g}\:\text{n}\text{o}\text{v}\text{e}\text{l}\:\text{r}\text{a}\text{t}\:2\:-\:\text{t}\text{i}\text{m}\text{e}\:\text{e}\text{x}\text{p}\text{l}\text{o}\text{r}\text{i}\text{n}\text{g}\:\text{f}\text{a}\text{m}\text{i}\text{l}\text{i}\text{a}\text{r}\:\text{r}\text{a}\text{t}\:}{\text{T}\text{i}\text{m}\text{e}\:\text{e}\text{x}\text{p}\text{l}\text{o}\text{r}\text{i}\text{n}\text{g}\:\text{n}\text{o}\text{v}\text{e}\text{l}\:\text{r}\text{a}\text{t}\:2\:+\:\text{t}\text{i}\text{m}\text{e}\:\text{e}\text{x}\text{p}\text{l}\text{o}\text{r}\text{i}\text{n}\text{g}\:\text{f}\text{a}\text{m}\text{i}\text{l}\text{i}\text{a}\text{r}\:\text{r}\text{a}\text{t}\:}$$

After completing the entire trial, the chambers and wire cages were cleaned with 70% ethanol. The movement of animals and the duration of stay of each subject in all 3 compartments were recorded using Any-Maze video tracking software (Version 7.1, Stoelting Co, Illinois, USA) linked with an overhead camera located in the sound isolated room where the test was carried out (Kim et al. 2019; Habib et al. 2023).

#### Morris Water Maze Test

Memory and spatial learning are most commonly investigated using the Morris water maze test. The maze consisted of a large circular pool with dimensions of 60 cm high and 200 cm in diameter which was divided into four almost equal quadrants and filled with water to a depth of 40 cm. The water was kept at room temperature (25 ± 1ºC) and made opaque using dark blue non-toxic water-soluble watercolor paint. A movable escape platform (15 cm x 15 cm) was submerged in the water in the center of a specific quadrant. The pool was placed in a low-lit room together with fixed distal visual clues that served as directional reference key for the rats to locate the target. A CCD camera suspended above the pool center recorded the swim paths of the animals and video output was digitized by Any-Maze video tracking software (Version 7.1, Stoelting Co, Illinois, USA). The water maze tests include 3 periods: initial spatial training, spatial reversal training and probe test.

- **Initial spatial training**: In this phase, rats were exposed daily to 2 training sessions of 2 min each with 10 min of inter-trial intervals for 4 consecutive days. During every training session, the animals were left free to find the hidden platform in the target quadrant. If the rat was successful in finding the hidden platform located at the NE quadrant during the designated time, it was allowed to stay on the platform for 10 s. However, if the animal failed to detect the platform during the allocated time, it was gently guided to reach the platform and was placed on it for 30 s. The average of the total time taken by the rat to locate the platform (escape latency) in the two training sessions of each day was calculated and was used as an indication for the spatial learning progress.
- **Spatial reversal training**: During reversal learning, the hidden platform was moved to the opposite quadrant (in this case from NE to SW). Reversal learning took 3 additional days with 2 trials/day, with procedures similar to initial training.
- **Probe test**: Twenty-four hours after the final reversal training trial, a probe test was performed where rats were returned to the pool from a new drop point after removing the platform and were allowed to explore the maze for 2 min during which their swim path and the time spent by each animal in the platform area was recorded and used as an indication for the reference memory (Dong et al. 2013).

### Brain Processing

After the behavioral tests, rats were sacrificed by decapitation under anesthesia with thiopental sodium (50 mg/kg i.p.) (El-Sahar et al. 2021). Brains were rapidly removed and washed with ice-cold saline. Then, the brains were randomly designated into three subsets. In the first subset of samples (*n* = 3/group), brains were fixed in 10% (v/v) buffered formalin for 72 h to undergo hematoxylin and eosin (H&E) staining for the histopathological examination and the immunohistochemical analysis of the prefrontal cortex. In the second and third subset of samples, prefrontal cortices were properly separated, flash-frozen in liquid nitrogen, and kept at − 80 °C for future biochemical tests. The prefrontal cortices of the second subset (*n* = 6/group) were used for ELISA quantification, while those of the third subset (*n* = 3/group) were used for both western blotting and qPCR analysis.

### Biochemical Parameters

#### Enzymelinked Immunosorbent Assay (ELISA)

The prefrontal cortex samples were homogenized in ice-cold saline to prepare 10% (w/v) homogenates that were used for the measurement of the biochemical markers using rat-specific ELISA assay kits in accordance with the manufacturer’s instruction. Neuronal nitric oxide synthase (nNOS) and caspase 12 using ELISA kits were quantified using kits provided by MyBioSource, Inc., San Diego, CA, USA (cat# MBS164640 and MBS260913, respectively). ELISA kits obtained from LifeSpan BioSciences, Inc., Seattle, WA, USA were utilized for the determination of postsynaptic density protein-95 (PSD-95), B-cell lymphoma 2 protein associated X-protein (Bax) and GAD67 (cat#LS-F6865, LS-F5064 and LS-F33015, respectively). Tumor necrosis factor-α (TNF-α) (cat# CSB-E11987r, Cusabio Co. Ltd, USA) and B-cell lymphoma/leukemia 2 protein (Bcl-2) (cat# abx155248, Abbexa Ltd, USA) were quantified using their corresponding kits. The results were expressed as pg/mg protein for Bax, Bcl-2 and TNF-α, ng/mg protein for caspase-12 and PSD-95 and mIU/mg protein for nNOS, where the protein content of tissue homogenate was determined by Bio-Rad protein assay kit, CA, USA, according to the method formerly described (Bradford 1976).

#### Western Blot

Parvalbumin, IRE-1, PERK, and ATF-6 protein expressions were determined by Western blot technique. In brief, the dissected prefrontal cortical tissues were homogenized, and their protein content was measured using the Bradford protein assay kit (Thermo Fisher Scientific Inc., MA, USA) according to Bradford’s method (Bradford 1976). Proteins of each sample were separated via sodium dodecyl sulfate polyacrylamide gel electrophoresis gel (SDS-PAGE), and then they were transferred into the nitrocellulose membrane. Tris-buffered saline with Tween 20 and 3% bovine serum albumin was used to block the non-specific binding sites. After that, the blocked blots were incubated at 4^◦^C overnight with 1:1000 dilution of the following primary antibodies against: Parvalbumin (cat# NB120-11427, NOVUS, Toronto, Canada), ATF-6 (cat# SAB2100170, Sigma-Aldrich, St. Louis, MO, USA), PERK (cat# P0074, Sigma-Aldrich, St. Louis, MO, USA), IRE-1 (cat#ZRB1072-4 × 25UL, Sigma-Aldrich, St. Louis, MO, USA), and β-actin (cat# SAB5600204, Sigma-Aldrich, St. Louis, MO, USA). After washing the blots multiple times in Tris-buffered saline with Tween 20, they were incubated with peroxidase-labelled secondary antibodies at room temperature for 1 h. An enhanced chemiluminescence substrate reaction (Amersham Biosciences, Arlington Heights, IL, USA) was used to obtain protein bands. Their corresponding intensities were measured using densitometric analysis using a scanning laser densitometer (Biomed Instrument, Inc., CA, USA). The results were expressed as arbitrary units relative to the intensity of the equivalent β-actin bands.

#### Quantitative RT-PCR

Total RNA was extracted from the prefrontal cortical tissues using total RNA extraction kit (cat#AM1830, Thermo Fisher Scientific, USA). The purity of the isolated RNA was determined spectrophotometrically by 260/280 nm with BioSpectrometer (Eppendorf AG, Germany). The reverse transcription of the extracted RNA into cDNA was developed using Reverse Transcription System High-Capacity cDNA Reverse Transcription Kit (cat #K4374966, Thermo Fisher Scientific, USA) in agreement with the kit’s guidelines. The gene expression of NMDAR subunit GluN2B (encoded by the GRIN2B gene) was assessed via quantitative real-time PCR using SYBR Green Universal PCR Master Mix (Applied Biosystems, CA, USA) following the manufacturer’s instructions. Table 1 shows the sequences of primers used. PCR conditions were set according to the standard procedure (50 °C for 2 min, 95 °C for 10 min followed by 40 cycles of 95 °C for 15 s and 60 °C for 60 s). The relative expression of target genes was acquired using the 2 − ΔΔCT formula using β-actin as a housekeeping gene.

**Table 1:** Sequence of primers, annealing temperatures and product sizes for PCR amplification

Subunit	5′-3′ Primer Sequence
NMDAR	Forward: CGCAGCGTGAGCCTGAA Reverse: CTCAAACATATGGGCGTAGGG
β-actin	Forward: AAGATCCTGACCGAGCGTGG Reverse: CAGCACTGTGTTGGCATAGAGG

### Histopathological Examination

Brains were flushed and fixed in 10% buffered formalin for 48 h. Then, samples were processed in serial ascending grades of ethanol, cleared in xylene, infiltrated, and embedded into Paraplast plus tissue embedding media. After that, 5 μm thick serial sagittal brain sections were cut by rotatory microtome for the examination of prefrontal cortical regions in different samples. Tissue sections were stained by hematoxylin and eosin (H&E) as a general staining method for microscopy. All standard procedures for samples fixation and staining were carried out according to (Culling 2013). All light microscopic investigation and data were obtained using Leica Application module for histological analysis attached to Full HD microscopic imaging system (Leica Microsystems GmbH, Germany). The histopathological examination was exerted by an expert investigator who was blinded to the identity of the samples.

### Immunohistochemistry

Tissue sections were cut and used for ionized calcium-binding adapter molecule-1 (Iba-1) immunostaining according to the method previously described (Özevren et al. 2020). Briefly, sections were deparaffinized and rehydrated, and then subjected to heat-induced antigen retrieval. Antigen retrieved brain tissue sections were treated by 0.3% hydrogen peroxide for blocking endogenous peroxidase for 15 min. After that, tissue was incubated with the primary antibody ant-Iba-1 (cat# ab108539, Abcam Limited, Cambridge, UK; 1:100 dilution) overnight at 4ºC. After multiple washing steps using PBS, the tissue slides were incubated with biotinylated secondary antibody Donkey Anti-Rabbit IgG (Dα-Rabbit HRP) (cat# 711 − 035–152; Jackson Immuno Research; 1:450 dilution) for 2 h at room temperature followed by another series of washing with PBS. Diaminobenzidine was used as a chromogen and added to the sections for up to 10 min. Tissue sections were then counterstained with hematoxylin, dehydrated, and cleared in xylene. Then, tissue slices were cover slipped and observed under a microscope by a blinded experienced histologist. According to (El-Deeb et al. 2022) using full HD microscopic imaging system and Leica Application system modules for histological analysis (Leica Microsystems GmbH, Wetzlar, Germany), at least six random non overlapping fields were scanned, segmented and analyzed for determining the relative mean positive area percentage of immunohistochemical expression levels of Iba-1 reactive microglia in the prefrontal cortex regions of each immuno-stained tissue section.

### Statistical Analysis

All results obtained were analyzed using one-way ANOVA or two-way ANOVA followed by Tukey’s multiple comparisons test and expressed as mean ± SD. Statistical analysis was performed using GraphPad Prism software (Version 9, San Diego, California, USA); a probability level of < 0.05 was accepted as statistically significant in all statistical tests.

## Results

### Fluvoxamine Improved ketamine-induced Impairment of Spatial Learning and Cognitive Flexibility

**Fig. 2:**
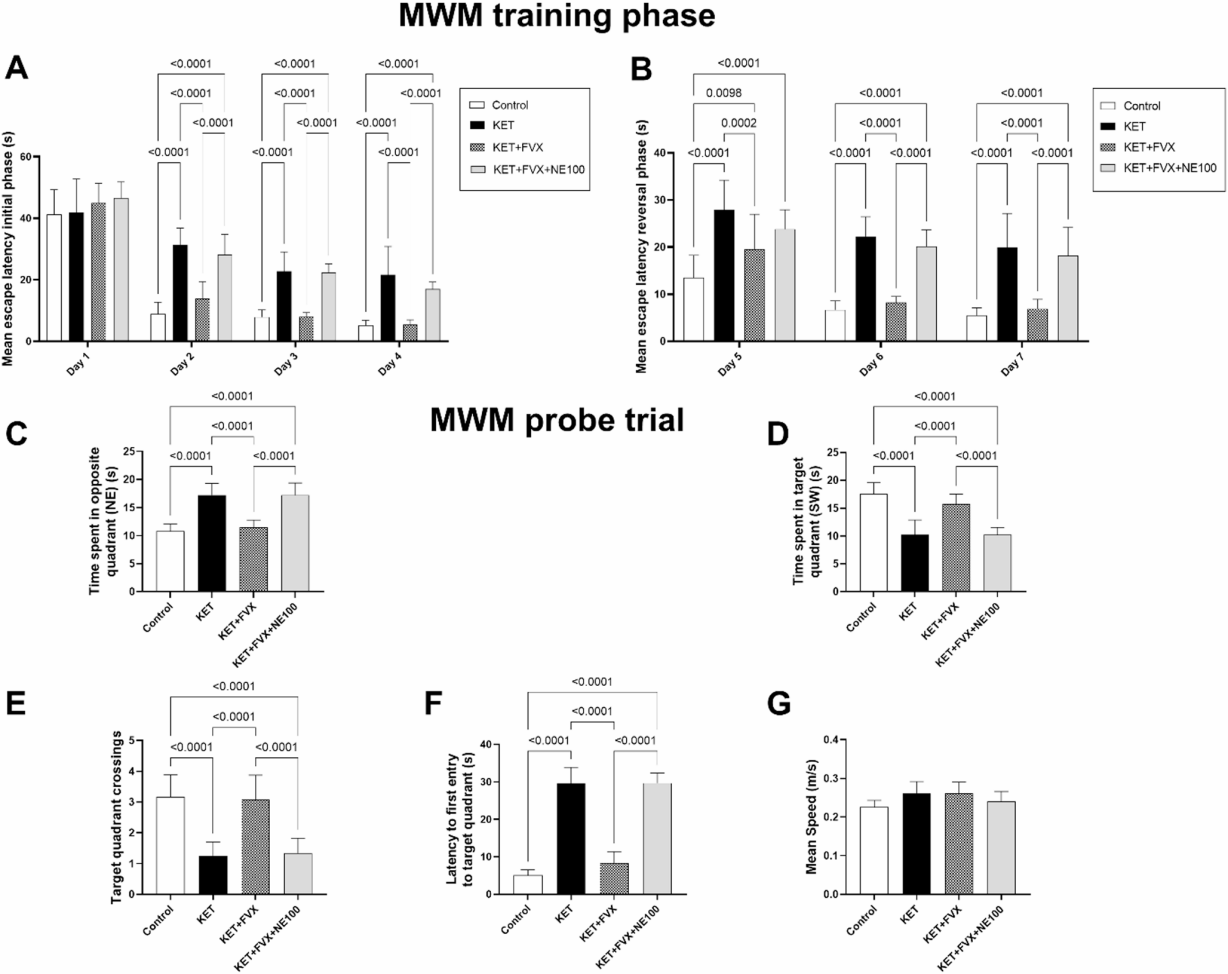
Effect of fluvoxamine on ketamine-induced spatial learning and cognitive flexibility dysfunction in MWM test (*n* = 12). **(A)** Escape latency in initial training phase target, **(B)** Escape latency in reversal training phase, **(C)** Time spent in target quadrant, **(D)** Time spent in opposite quadrant, **(E)** Latency to first entry to target quadrant, **(F)** Target quadrant crossings, and **(G)** Mean speed. Data were expressed as mean ± SD using one-way ANOVA followed by Tukey’s multiple comparison analysis, *p* < 0.05, except for mean escape latency in both initial training and reversal training phases, two-way ANOVA was used followed by Tukey’s multiple comparisons test. *KET* ketamine, *FVX* fluvoxamine

Rats were examined in MWM task to assess their cognitive abilities and cognitive flexibility as verified by time spent in target quadrant (SW) (F (3, 44) = 44.70; *P* < 0.0001), time taken in opposite quadrant (NE) (F (3, 44) = 48.03; *P* < 0.0001), latency for the first entry to target quadrant (F (3, 44) = 228.3; *P* < 0.0001), and number of entries to the target quadrant (F (3, 44) = 33.87; *P* < 0.0001). During the first day of the initial training phase, no significant difference in the mean escape latency was noticed among the experimental groups, while a significant decrease was observed in the KET + FVX group by 55, 64 and 75% in day 2, day 3 and day 4, respectively, as compared to the KET group (Fig. 2A). Upon switching the platform position in the reversal training phase, the mean escape latency decreased significantly in the KET + FVX group by 30, 63, and 65% in day 5, day 6 and day 7, respectively, as compared to the KET group which showed no cognitive flexibility to the platform position change (Fig. 2B). In the probe test, KET-subjected group spent significantly lower time in SW quadrant (41%) and almost halved the number of entries to target quadrant, showing reduced special learning and cognitive flexibility, as compared to the control group, together with retarded entrance to target quadrant (4.8-fold), while increased the searching time for the platform in the opposite quadrant (59%). Treatment with fluvoxamine in KET + FVX group significantly recovered the learning abilities and cognitive flexibility as demonstrated by an increase in time spent in the target quadrant and entries number by 54% and 1.4-fold, respectively, while lessened the latency to firstly enter the target quadrant and their spent time in the opposite quadrant by 71 and 32%, respectively, as compared to the untreated rats. However, KET + FVX + NE100 group dampened fluvoxamine’s cognition-ameliorating effect as indicated by the delayed entrance to target quadrant (2.5-fold), and the increased navigation in the opposite quadrant (50%), while decreasing the time in target quadrant (*P* < 0.0001) along with the number of target quadrant crossings by 35 and 56.7%, respectively, when compared to the fluvoxamine-treated rats (Fig. 2C-F). There was no significant difference found between the experimental groups in the mean speed (F (3, 44) = 2.186; *P* = 0.1031) (Fig. 2G).

### Fluvoxamine Counteracted Ketamine-induced Deterioration of Social Interaction

**Fig. 3:**
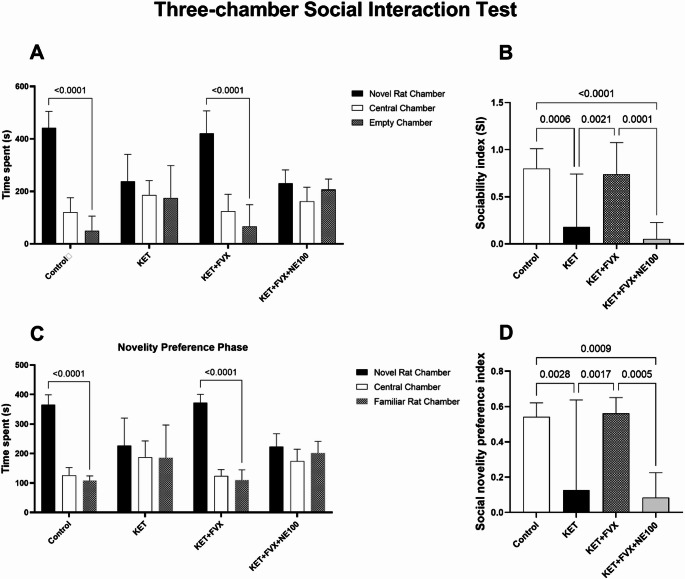
Effect of fluvoxamine on ketamine-induced social interaction deterioration in the three-chamber sociability test (*n* = 12). **(A)** Time spent in each chamber in the sociability phase. **(B)** Sociability index. **(C)** Time spent in each chamber in the social novelty preference phase. **(D)** Sociability preference index. All data are presented as a mean ± SD using one-way ANOVA followed by Tukey’s multiple comparison analysis, *p* < 0.05, except for time spent in each chamber in both sociability and social novelty preference phases, two-way ANOVA was used followed by Tukey’s multiple comparisons test. *KET* ketamine, *FVX* fluvoxamine

The KET group showed impairment of social interaction evidenced by profound decline in SI (F (3, 44) = 13.8; *P* < 0.0001) and SNI (F (3, 44) = 10.87; *P* < 0.0001), by 77% and 76%, respectively, in contrast to the control group. Interestingly, fluvoxamine was able to improve the sociability functions of fluvoxamine-treated rats as shown by a considerable increase in SI and SNI by 3- and 3.4-folds, respectively, as compared to the KET group. However, concurrent administration of NE-100 with fluvoxamine abolished fluvoxamine’s sociability enhancing effect causing remarkable reduction in SI and SNI by 92.86% and 84.9%, respectively, in contrast to the KET + FVX group, (Fig. 3).

### Fluvoxamine Lessens Ketamine-induced Apoptosis and Inflammation in Prefrontal Cortex

**Fig. 4:**
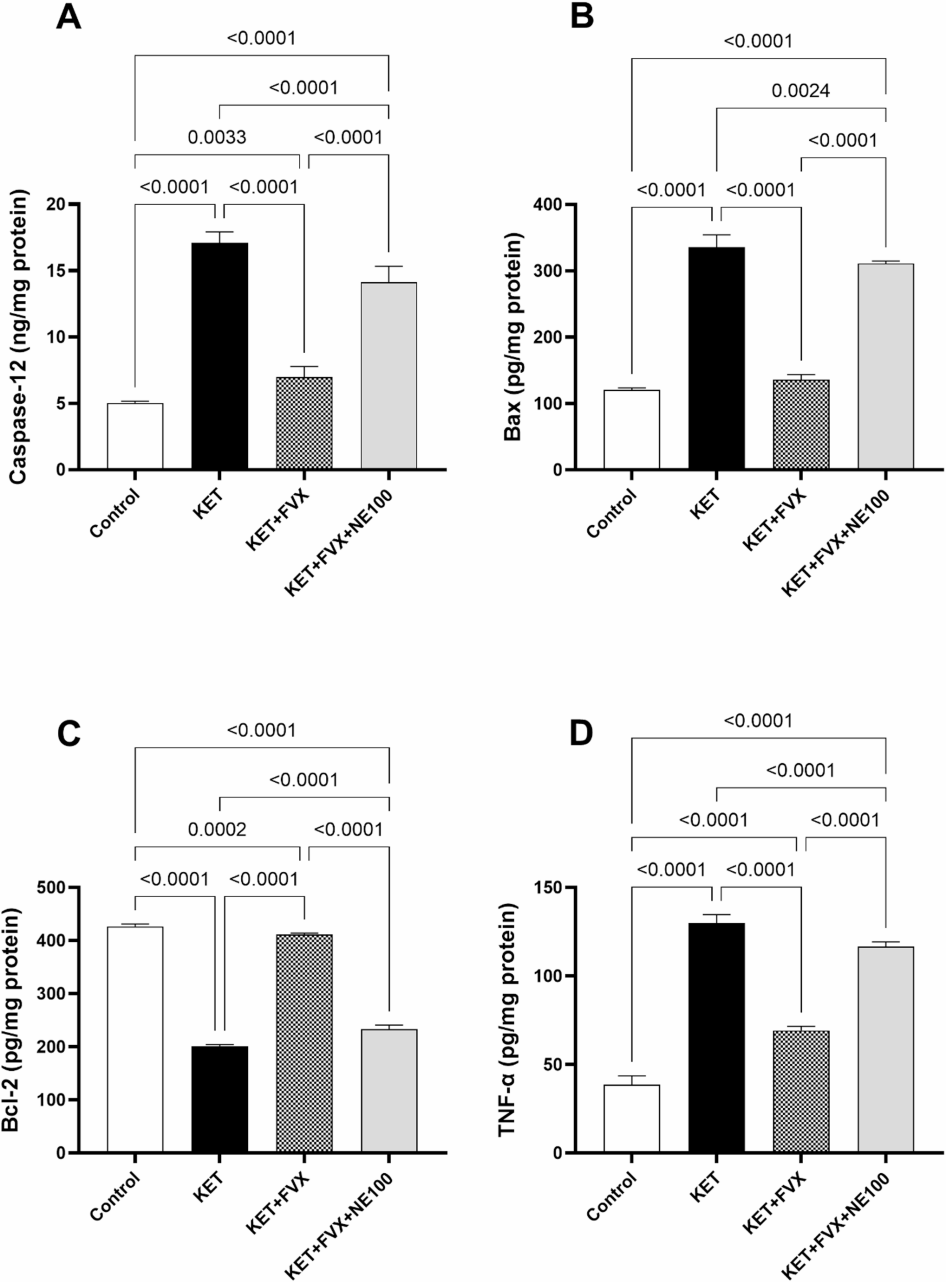
Effect of fluvoxamine on ketamine-induced changes of the prefrontal cortical contents (*n* = 6) of **(A)** caspase-12, **(B)** Bax, **(C)** Bcl-2, and **(D)** TNF-α. All data are presented as a mean ± SD using one-way ANOVA followed by Tukey’s multiple comparison analysis, *p* < 0.05. *KET* ketamine, *FVX* fluvoxamine

Ketamine model of psychosis was associated with a profound state of apoptosis and inflammation. This was evidenced by significantly increasing the prefrontal cortical contents of the apoptotic markers; caspase-12 and Bax by 2.4 and 1.7 folds, respectively, as well as the inflammatory marker TNF-α by 2.36 folds, in contrast to declined concentration of the anti-apoptotic marker Bcl-2 by 52.8% as compared to the control group (F (3, 20) = 281.3 for caspase-12, 733.9 for Bax, 696.1 for TNF-α and 3509 for Bcl-2, *p* < 0.0001). Contrastingly, fluvoxamine displayed anti-apoptotic and antioxidant effects, as indicated by the suppression of caspase-12 and Bax levels as well as TNF-α by 59.19, 59.5, and 46.75%, respectively, contrary to the elevation of Bcl-2 content by 1-fold compared to the KET group. The coadministration of NE100 hampered the observed actions of fluvoxamine consequently triggering a significant rise in the apoptotic markers; caspase-12 and Bax by 1- and 1.2-folds, respectively, along with augmented TNF-α level (68.4%), while dropping that of Bcl-2 (43.3%), compared to the KET + FVX group (Fig. 4).

### Fluvoxamine Mitigates Ketamine-induced Inhibition of nNOS/PSD-95/NMDAR Signaling Pathway

**Fig. 5:**
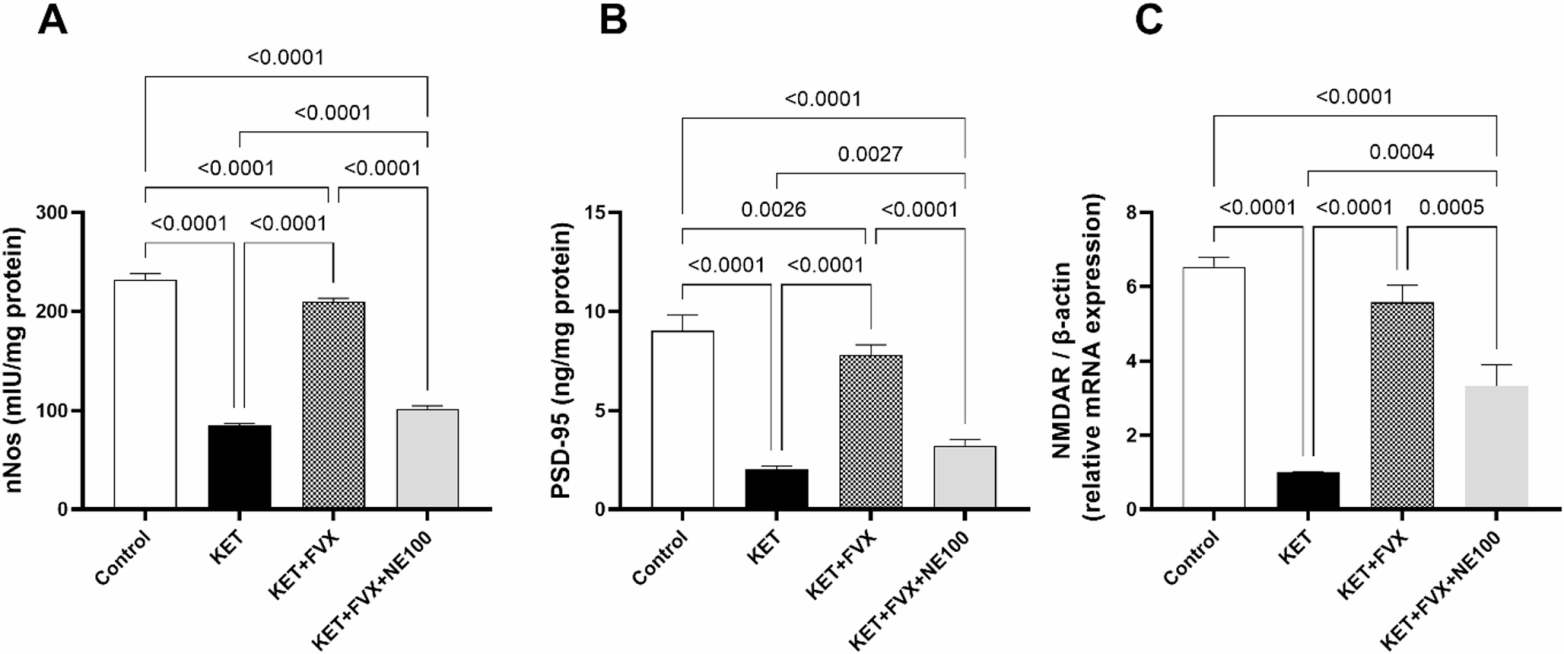
Effect of fluvoxamine on ketamine-induced changes of the prefrontal cortical contents (*n* = 6) of **(A)** nNOS, and **(B)** PSD-95 protein expression as well as **(C)** NMDAR gene expression (*n* = 3). All data are presented as a mean ± SD using one-way ANOVA followed by Tukey’s multiple comparison analysis, *p* < 0.05. *KET* ketamine, *FVX* fluvoxamine, *NMDAR* n-methyl-d-aspartate receptor

Ketamine injection provoked definite depression of the prefrontal cortical contents of nNOS and PSD-95, as well as NMDAR mRNA expression by 63.18, 77.5 and 84.6% compared with the control values, respectively; F (3, 20) = 1981 (nNOS), 279.6 (PSD-95), and F (3, 8) = 121.9 for NMDAR, p *<* 0.0001. Such depression was mitigated by fluvoxamine treatment producing considerable augmentation in nNOS, PSD-95, and NMDAR levels by 1.45-, 2.86- and 4.53-folds, respectively, as related to the KET group. Concurrent administration of NE100 alleviated the effect of fluvoxamine on these biomarkers leading to a pronounced reduction in their levels by 1-fold (nNOS), 58.8% (PSD-95), and 40.1% (NMDAR), when compared to the KET + FVX group (Fig. 5).

### Fluvoxamine Attenuates ketamine-induced Disruption of Glutamatergic Neurotransmission and Activation of parvalbumin-positive GABAergic Interneurons

**Fig. 6:**
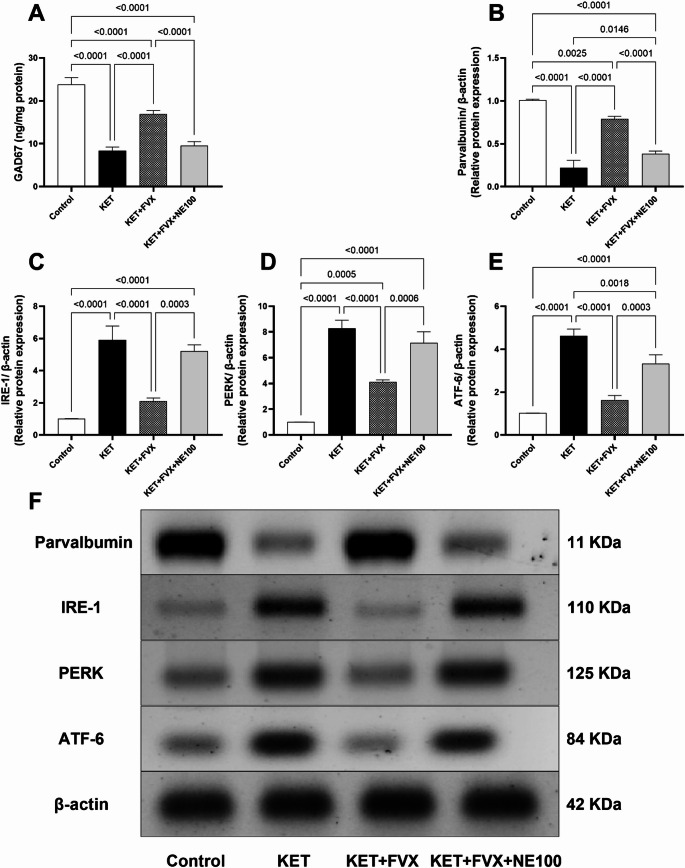
Effect of fluvoxamine on ketamine-induced changes of prefrontal cortical contents (*n* = 3) of **(A)** GAD67, **(B)** parvalbumin, **(C)** IRE-1, **(D)** PERK, and **(E)** ATF-6 protein expression as well as **(F)** their representative blots. All data are presented as a mean ± SD using one-way ANOVA followed by Tukey’s multiple comparison analysis, *p* < 0.05. *KET* ketamine, *FVX* fluvoxamine

The prefrontal cortical protein levels of the biomarkers parvalbumin and GAD67, which are indicative of parvalbumin GABAergic interneurons activation, were significantly diminished in the KET group animals by 78.2 and 64.7%, respectively, as compared to the control group; F (3, 8) = 166.0 and F (3, 20) = 256.2, respectively, p *<* 0.0001. Treatment of ketamine-subjected rats with fluvoxamine suppressed such depletions resulting in clear augmentation in their protein levels by 2.59 and 1 folds, respectively, versus the KET group. Fluvoxamine-instigated rise in these parameters was hindered by concurrent administration of NE100 leading to a remarkable drop in their levels by 51% (parvalbumin) and 43% (GAD67), as compared to KET + FVX group (Fig. 6A and B).

### Fluvoxamine Lessens Ketamine-Induced Activation of ER Stress

The protein expressions of ER stress sensors which are IRE-1, PERK and ATF-6 in the prefrontal cortex were significantly increased in KET group animals by 4.8-, 7.2- and 3.5- folds, respectively, when compared to the control group; F (3, 8) = 69.92, 109.5, and 108.0, respectively, *p* < 0.0001. Fluvoxamine treatment mitigated these effects inducing a marked depletion in these biomarkers’ levels by 64 (IRE-1), 50 (PERK) and 65% (ATF-6), as compared with the KET group. Fluvoxamine-induced drop in IRE-1, PERK and ATF-6 protein contents was tackled by concurrent administration of NE100 by 1.4-folds, 73% and 1-fold, respectively, as compared to KET + FVX group (Fig. 6C-F).

### Fluvoxamine Attenuates Ketamine-Induced Immunohistochemical Changes in Rats

**Fig. 7:**
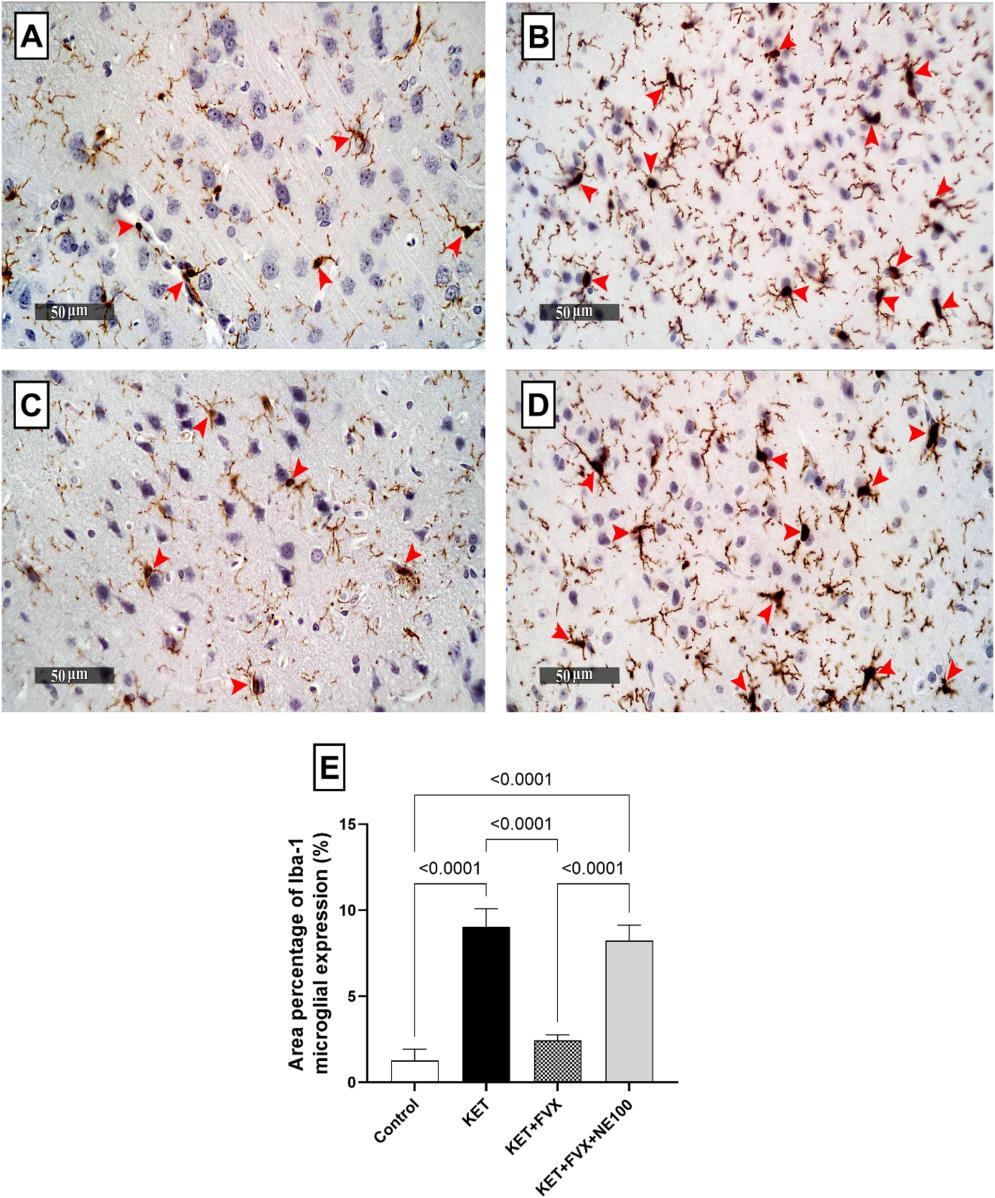
Fluvoxamine attenuated ketamine-induced immunohistochemical alterations in the prefrontal cortical level of Iba-1. Photomicrographs (*n* = 6) of **(A)** control group, **(B)** KET group, **(C)** KET + FVX group, and **(D)** KET + FVX + NE-100 group. The illustrated photos were magnified by × 400, with scale bars = 50 μm. (**E**) A bar chart representing the relative mean positive area percentage of immunohistochemical expression levels of Iba-1 reactive microglia in prefrontal cortex in each group. All data are presented as a mean ± SD using one-way ANOVA followed by Tukey’s multiple comparison analysis, *p* < 0.05. *KET* ketamine, *FVX* fluvoxamine

The immunohistochemical investigation of Iba-1 which is an indicative of the inflammatory response caused by microglial activation in prefrontal cortex was carried out. The KET group showed significant increase in immunoreactivity of Iba-1 by 6.1 folds, when compared to the control group; F (3, 20) = 149.3, *p* < 0.0001. Treatment with fluvoxamine suppressed such elevation inducing a marked drop in the biomarker level by 73% as compared with KET group. Fluvoxamine-induced rise in this parameter was hindered by concurrent administration of NE100 leading to a distinct elevation of 2.3 folds, as compared to KET + FVX group (Fig. 7).

### Fluvoxamine Attenuates Ketamine-Induced Cortical Histopathological Changes

**Fig. 8:**
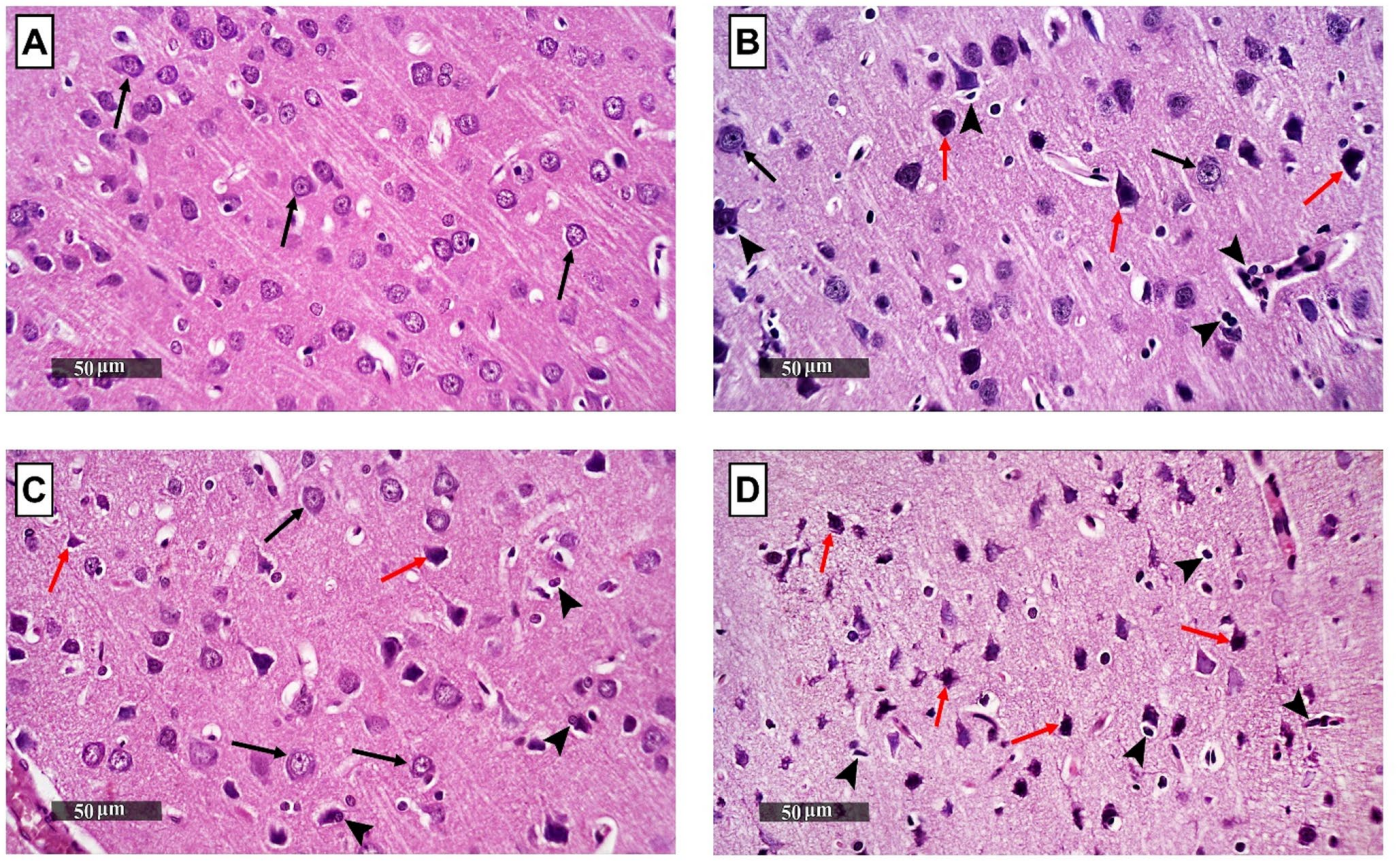
Fluvoxamine attenuated ketamine-induced histopathological changes in the prefrontal cortex. Representative H&E photomicrographs of the prefrontal cortex sections (*n* = 3) of **(A)** control group, **(B)** KET group, **(C)** KET + FVX group, and **(D)** KET + FVX + NE-100 group. Intact neurons were exemplified by *black arrows* whereas the degenerated ones were represented by *red arrows*. *Arrowheads* indicated glial cells infiltrates. The illustrated photos were magnified by × 400, with scale bars = 50 μm. *KET* ketamine, *FVX* fluvoxamine

Representative H&E photomicrographs of the prefrontal cortex from different brain samples of all experimental groups were microscopically examined. The brain samples of the control group demonstrated normal histological structures of cerebral cortical layers with many apparent intact neurons all over different layers (black arrow) and minimal sporadic records of degenerative changes with intact intercellular brain tissue matrix and minimal glial cells infiltrates (Fig. 8A). The KET group brain samples showed wide areas of outer and deeper cortical layers with marked neuronal degenerative changes and nuclear pyknosis (red arrow) alternated with fewer apparent intact cells in middle and inner layers (black arrow). This was accompanied by mild edema of brain matrix and marked increase of reactive glial cells infiltrates including microglial cells together with multiple figures of neuronophagia (arrowhead) (Fig. 8B). Fluvoxamine treatment demonstrated moderate neuroprotective efficacy around the cortical layers as demonstrated by alternated figures of more apparent intact neurons (black arrow) and fewer abnormal degenerative changes (red arrow) along with significant fewer abnormal microglial infiltrates records (arrowhead) (Fig. 8C). The brain tissues of NE100-administered group showed almost comparable histopathological alterations with that detected in KET group samples (Fig. 8D).

## Discussion

The current study highlights the beneficial potential of fluvoxamine, a Sig-1R agonist, in modulating cellular abnormalities and behavioral deficits in schizophrenia. Its potential antipsychotic effect is attributed mainly to the stimulation of Sig-1R which modulates UPR, aiding in protein refolding and the restoration of ER homeostasis. It inhibited UPR-regulatory proteins and exerted anti-inflammatory and anti-apoptotic effects. It restored the glutamatergic neurotransmission and activated the parvalbumin-positive GABAergic interneurons ameliorating the cognitive deficits and the histopathological alterations in the prefrontal cortex.

The administration of subanesthetic doses of ketamine (30 mg/kg/day) for 5 consecutive days is a commonly used animal model for studying schizophrenia as it induces behavioral changes simulating the positive, negative, and cognitive symptoms of schizophrenia (Nawwar et al. 2022; Ngubane et al. 2024). Herein, fluvoxamine counteracted ketamine-induced social deficits as demonstrated by increased sociability and social novelty indices. In line with this study, Sig-1Rs modulators such as SOMCL-668, were found to improve social and cognitive function in chronic phencyclidine-induced schizophrenia (Chen et al. 2022). Hashimoto and colleagues investigated the effect of SSRIs, including fluvoxamine, on phencyclidine (PCP)-induced cognitive impairments in mice revealing that sub-chronic administration of fluvoxamine significantly improved cognitive impairment in the novel object recognition test. This therapeutic effect of fluvoxamine, as well as the Sig-1R agonists SA4503 and DHEA-S, was inhibited by the Sig-1R antagonist NE-100, implying that Sig-1R activation might underlie the cognitive benefits and represent a potential target for the treatment of cognitive deficits in schizophrenia (Hashimoto et al. 2007). In the present work, fluvoxamine also improved spatial learning and cognitive flexibility of ketamine-subjected rats in MWM. NMDARs appear to be involved in the flexible cognitive processes that allow reversal learning in spatial tasks such as MWM (Thonnard et al. 2019). Blocking NMDARs with non-competitive antagonists has been found to hinder cognitive flexibility in reversal learning phase (Lobellova et al. 2013). Interestingly, Sig-1R agonists have gained interest for their role in cognitive impairment and neuroprotection (Malar et al. 2023). It has been reported that fluvoxamine enhanced spatial working memory and alleviated cognitive impairments in schizophrenia patients (Niitsu et al. 2012; Martin et al. 2021). These behavioral improvements observed with fluvoxamine treatment were abolished in the NE-100 co-treated group.

Besides the behavioral alterations, ketamine injection was accompanied by marked histopathological changes which were attenuated by fluvoxamine treatment affording neuroprotection. Previous studies have documented that rat model of ketamine-induced schizophrenia showed morphological and functional changes in the prefrontal cortex (Homayoun and Moghaddam 2007; Gonzalez-Burgos and Lewis 2012). The prefrontal cortex is an essential brain area for cognitive flexibility (Stefani and Moghaddam 2010; Park and Moghaddam 2017), social cognition, and spatial memory retrieval (Jo et al. 2007; Bicks et al. 2015). Patients with prefrontal cortex dysfunction reported deficits in spatial working memory performance (Friedman and Robbins 2022).

Herein, fluvoxamine amended ketamine’s inhibitory effect on parvalbumin and GAD67, which are biomarkers of parvalbumin GABAergic interneuron’s function. Parvalbumin is a calcium-binding protein highly expressed in a subset of GABAergic interneurons (Druga et al. 2023). These parvalbumin-positive interneurons play a crucial role in regulating gamma oscillations (30–80 Hz), which are essential for cognitive processes such as working memory and attention (Milicevic et al. 2024). GAD67, encoded by the GAD1 gene, is an enzyme responsible for the synthesis of GABA, the primary inhibitory neurotransmitter in the brain, and is widely expressed in inhibitory interneurons, including parvalbumin-positive cells (Lewis and Hashimoto 2007; Magri et al. 2018). The co-expression of both parvalbumin and GAD67 in inhibitory interneurons is critical for their proper functioning (Marín 2012). According to the literature, while the number of parvalbumin-expressing neurons remains constant, there is a considerable drop in parvalbumin expression among these neurons in patients with schizophrenia (Lewis et al. 2012). Likewise, decreased levels of GAD67 mRNA and protein are consistently reported in the postmortem brains of schizophrenia patients (de Jonge et al. 2017; Fujihara 2023), with the reduction being most pronounced in parvalbumin-positive interneurons, suggesting a functional impairment in these cells (Hashimoto et al. 2009). The reduction of GAD67 likely leads to reduced GABA synthesis and compromised inhibitory control, contributing to the excitatory-inhibitory imbalance seen in schizophrenia (Xu and Wong 2018). In addition to its uncompetitive inhibitory effects on NMDAR, ketamine administration during late adolescence in rats was reported to exhibit a reduction of GABAergic transmission, accompanied by a reduced number of parvalbumin and GAD67 positive cells and reduced immunoreactivity for both proteins (Jeevakumar et al. 2015; Zorumski et al. 2016).

Normally, NMDAR activation triggers Ca^2+^ influx and regulates other signaling pathways, including nNOS. Activation of nNOS via NMDARs requires contact with the scaffold protein PSD-95, resulting in an NMDAR/PSD-95/nNOS complex (Doucet et al. 2012). This association plays a key role in a range of normal neuronal activities, including synaptic plasticity, learning, and memory, while direct inhibition nNOS, PSD-95 or NMDARs can have severe side effects, contributing in the development of brain pathologies such as schizophrenia (Coley and Gao 2018; Gu and Zhu 2020). nNOS adaptor protein (NOS1AP), originally reported as a competitor of PSD-95/nNOS interaction, is now considered an inhibitor of NMDAR-driven nNOS function (Courtney et al. 2014). It is a promising candidate for a schizophrenia susceptibility gene owing to its ability to bind to nNOS. An excess of NOS1AP can impede the interaction between PSD-95 and nNOS, resulting in the disruption of NMDAR/PSD-95/nNOS signal transduction (Brzustowicz 2008; Matiiv et al. 2022). A decrease in frontal cortical nNOS and PSD-95 in schizophrenic patients causes a severe glutamatergic attenuation within the brain with consequent overall decrease in the prefrontal cortical neuronal activity inducing schizophrenic-like symptoms like cognitive deficits and working memory impairments (Nasyrova et al. 2015; Coley and Gao 2019). In consistence with the previous studies, ketamine administration induced reduction in prefrontal cortical contents of nNOS and PSD-95, as well as NMDAR mRNA expression contributing to spatial learning and cognitive flexibility impairment, reported herein. In contrast, several studies now show that NOS1AP can promote rather than inhibit NMDAR/nNOS-dependent responses, including excitotoxic signaling (Li et al. 2017). However, the notion of NOS1AP as a nNOS inhibitor dominates human disease genetics research (Courtney et al. 2014).

Protein misfolding and the production of toxic aggregates at the brain level, caused by dysregulation of proteostasis, are linked to a variety of neuropsychiatric disorders (Olivero et al. 2018). The ER is an important organelle that regulates protein quality and cellular homeostasis (Ghemrawi and Khair 2020). The buildup of unfolded or misfolded proteins inside the cell causes the failure of the ER to cope with the excess of protein load, resulting into ER stress and UPR (Uddin et al. 2020). The UPR is controlled by three ER transmembrane stress sensors: PERK, ATF-6, and IRE-1 (Gardner et al. 2013). These proteins have luminal domains that detect unfolded protein peptides, as well as cytosolic regions that activate signaling pathway(Kim 2024). Typically, these sensors are deactivated in the ER due to their link with glucose-regulated protein 78 (GRP78; also known as BiP) which gets dissociated from ER stress transducers upon UPR activation (Liu et al. 2023). In the current research, the KET group rats exhibited a marked increase in cortical protein expressions of ATF-6, PERK, and IRE-1; effects that were attenuated by treatment with fluvoxamine. The cytoplasmic protein kinase domain of PERK phosphorylates eIF2α, limiting protein production and relieving ER stress. The phosphorylation of eIF2α promotes stress-related mRNA translation by activating transcription factor 4 (ATF-4) (B’chir et al. 2013). The latter stimulates CHOP (C/EBP Homologous Protein) expression which in turn activates the transcription of ER stress chaperones that can regulate the shift from adaptation to neuronal cell survival by contributing to faulty protein refolding (Schröder 2008; Pitale et al. 2017). Moreover, upon prolonged unresolved ER stress, CHOP tends to induce apoptosis, through downregulating the expression of anti-apoptotic Bcl-2, while upregulating the expression of Bcl-2 Interacting Mediator of cell death (BIM), causing increased Bax expression (Hu et al. 2019). IRE-1 is an important sensor of ER stress that controls both adaptive and apoptotic pathways in UPR (Zhu et al. 2014). When activated by ER stress, IRE-1 undergoes oligomerization and autophosphorylation, resulting in endoribonuclease activity that splices X-box-binding protein 1 (XBP1) mRNA to create the active transcription factor sXBP1, which upregulates chaperone molecules involved in protein refolding and ER-associated degradation (ERAD), aiding in the restoration of ER homeostasis (Junjappa et al. 2018; Adams et al. 2019). However, with persistent or severe ER stress, IRE-1 switches to pro-apoptotic signaling. It interacts with TRAF-2 to activate JNK, hence promoting the mitochondrial malfunction and death (Bhattarai et al. 2021). JNK phosphorylation activates pro-apoptotic proteins such as Bax, while blocks anti-apoptotic Bcl-2 (Almanza et al. 2019). TRAF-2 also activates caspase-12 which subsequently cleaves and activates downstream executioner caspases, such as caspase-3, to induce apoptosis (Szegezdi et al. 2003; Di Sano et al. 2006; Ron and Walter 2007). Concerning ATF-6, when ER stress occurs, it travels to the Golgi apparatus and is broken by proteases releasing its active cytosolic form (Chen et al. 2023). ATF-6 can also contribute to apoptosis if ER stress is high and unresolved, by increasing the production of pro-apoptotic molecules such as CHOP (Bueter et al. 2009). Under ER stress, UPR activation can contribute to the inflammatory activation of microglia (Hasan et al. 2024). Persistent activation of UPR pathways, particularly through IRE-1 and PERK signaling, increases TNF-α production and contributes to brain inflammation (Bettigole and Glimcher 2015).

As chaperones, Sig-1Rs have the ability to modulate the UPR (Nguyen et al. 2015). Sig-1R combines with the ER chaperone and signaling regulator BiP/GRP78 forming an inactive complex. Upon activation under ER stress or in response to ligand stimulation, it separates from BiP/GRP78, and can alter the activity of other proteins such as PERK, IRE-1α, and ATF-6 (Mori et al. 2013). Herein, Sig-1R stimulation by fluvoxamine was associated with reduced cortical expression of PERK, IRE-1, and ATF-6 proteins with consequent decrease in cortical content of caspase-12, Bax and TNF-α, in contrast to elevating Bcl-2. Thus, Sig-1R stimulation contributes to neuronal protection from stress-induced damage and supporting synaptic function by maintaining proper protein folding and trafficking. In context, Sig-1R agonist PRE-084 ameliorated kidney injury in rat model of adenine-induced chronic kidney disease by decreasing the expression of ER stress proteins; PERK, ATF-6, and IRE-1α as well as reducing caspase-12 (Kumaran et al. 2023). Sig-1R antagonist BD-1047 abolished these effects (Shi et al. 2022). Additionally, fluvoxamine was found to mitigate protein misfolding in autism owing to the activation of Sig-1R (Mohamed et al. 2024). It is noteworthy that NE-100 pretreatment abrogated the cellular and behavioral effects afforded by fluvoxamine, confirming the role of Sig-1R in mediating fluvoxamine’s possible therapeutic effects in the current model of schizophrenia.

Despite the promising findings regarding the effects of fluvoxamine on ketamine-induced schizophrenia-like behaviors via Sig-1R activation, this study has some limitations that should be acknowledged. First, the ketamine-induced model, while widely used, does not fully capture the complexity or chronicity of schizophrenia as observed in humans, limiting its translational value. Future studies should explore the use of alternative and more etiologically relevant models of psychosis, including genetic models, to validate the present findings. Second, the exclusive use of male rats precludes any conclusions regarding sex-specific effects, which are increasingly recognized as important factor in neuropsychiatric research. Third, the study did not investigate the effects of NE-100 alone on ketamine-treated rats, leaving the specific role of Sig-1R blockade in this context unresolved and future work should evaluate its independent effects. Also, our study did not directly assess Sig-1R expression in relevant brain regions. Thus, future research should characterize the receptor levels across key areas such as the prefrontal cortex and hippocampus, and explore how these changes correlate with cognitive and social deficits in schizophrenia. Moreover, while previous research has shown that fluvoxamine– but not sertraline, an SSRI with Sig-1R antagonistic properties– ameliorates PCP-induced cognitive deficits in mice, the current study did not examine whether sertraline has similar or differing effects in the ketamine model, which could help clarify the role of Sig-1R modulation among SSRIs.

## Conclusion

In conclusion, the current study concludes that fluvoxamine, a Sig-1R agonist, can improve spatial learning and alleviate the primary symptoms of schizophrenia, including social deficits and cognitive inflexibility. Owing to its activation of molecular chaperone Sig-1R, fluvoxamine participated in protein refolding, reducing the UPR markers; PERK, IRE-1, and ATF-6. It dampened apoptosis and inflammation restoring ER homeostasis. It restored the glutamatergic neurotransmission and the function of parvalbumin-positive GABAergic interneurons. Thus, fluvoxamine can be considered as a prosperous candidate for amelioration of schizophrenia.

## Electronic Supplementary Material

Below is the link to the electronic supplementary material.

Supplementary Material 1

Supplementary Material 2

## Data Availability

All data are available upon request from the corresponding author.

## Abbreviations

ER: Endoplasmic reticulum
UPR: Unfolded protein response
NMDAR: N-methyl D-aspartate receptor
nNOS: Neuronal nitric oxide synthase
PSD-95: Postsynaptic density protein-95
GAD67: Glutamate decarboxylase 67
UPR: Unfolded protein response
IRE-1: Inositol-requiring enzyme type 1
PERK: Protein kinase R-like ER kinase
ATF-6: Activating transcription factor 6
Iba-1: Ionized calcium-binding adaptor molecule 1
TNF-α: Tumor necrosis factor-α
TRAF-2: Tumor necrosis factor (TNF) receptor associated factor-2
ASK1: Apoptosis signal-regulating kinase 1
JNK: c-Jun N-terminal protein kinase
eIF2α: Eukaryotic initiation factor 2α
Sig-1R: Sigma-1 receptor
SSRIs: Selective serotonin reuptake inhibitor
BiP: Binding immunoglobulin protein
NOS1AP: nNOS adaptor protein
SNPs: Single nucleotide polymorphism
GRP78: Glucose-regulated protein 78
CHOP: C/EBP homologous protein
XBP1: X-box-binding protein 1
BIM: Bcl-2 interacting mediator of cell death
