# Supplementary figures and images for: Sigma-1 Receptor Activation by Fluvoxamine Ameliorates ER Stress, Synaptic Dysfunction and Behavioral Deficits in a Ketamine Model of Schizophrenia
Source: J Neuroimmune Pharmacol. 2025 Jul 25;20(1):76. doi: 10.1007/s11481-025-10231-4 (PMC12296813; doi:10.1007/s11481-025-10231-4)

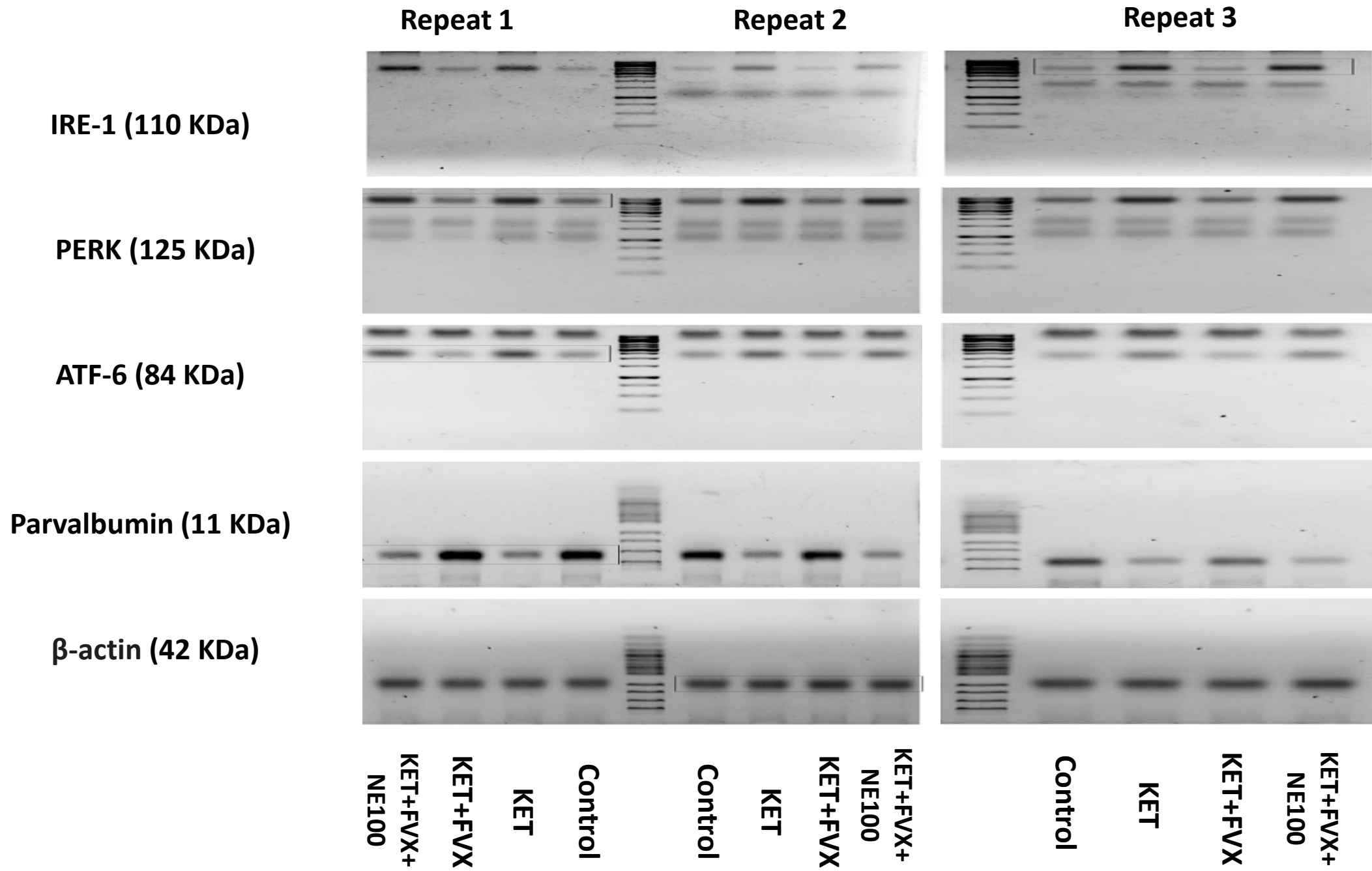

Supplement: Supplementary file 1 — Supplementary Material 1 [file 11481_2025_10231_MOESM1_ESM.pdf]

# MWM training phase

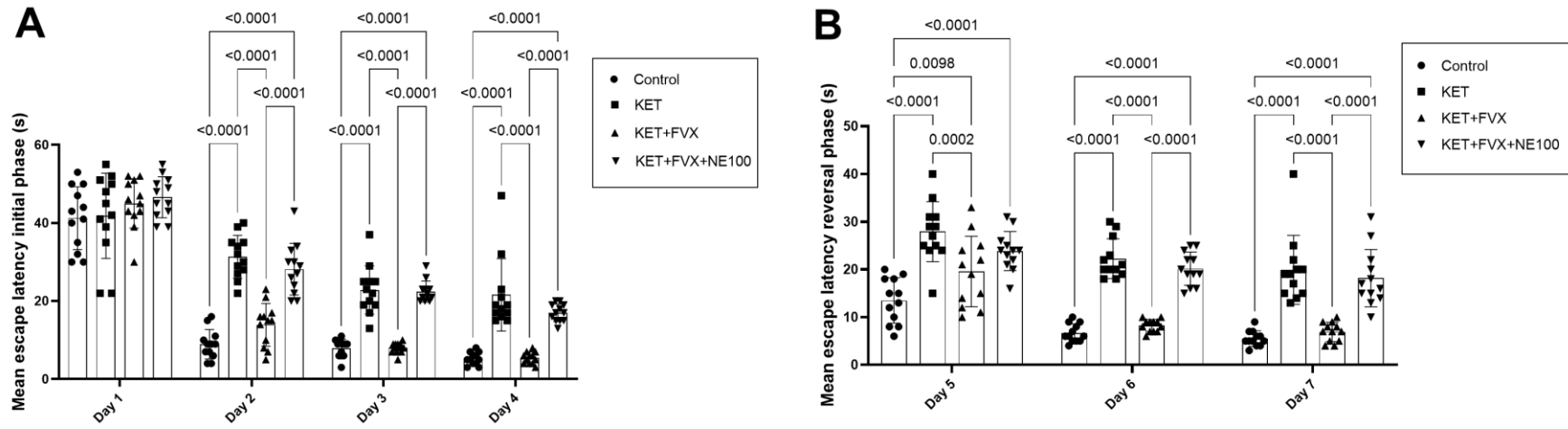

# MWM probe trial

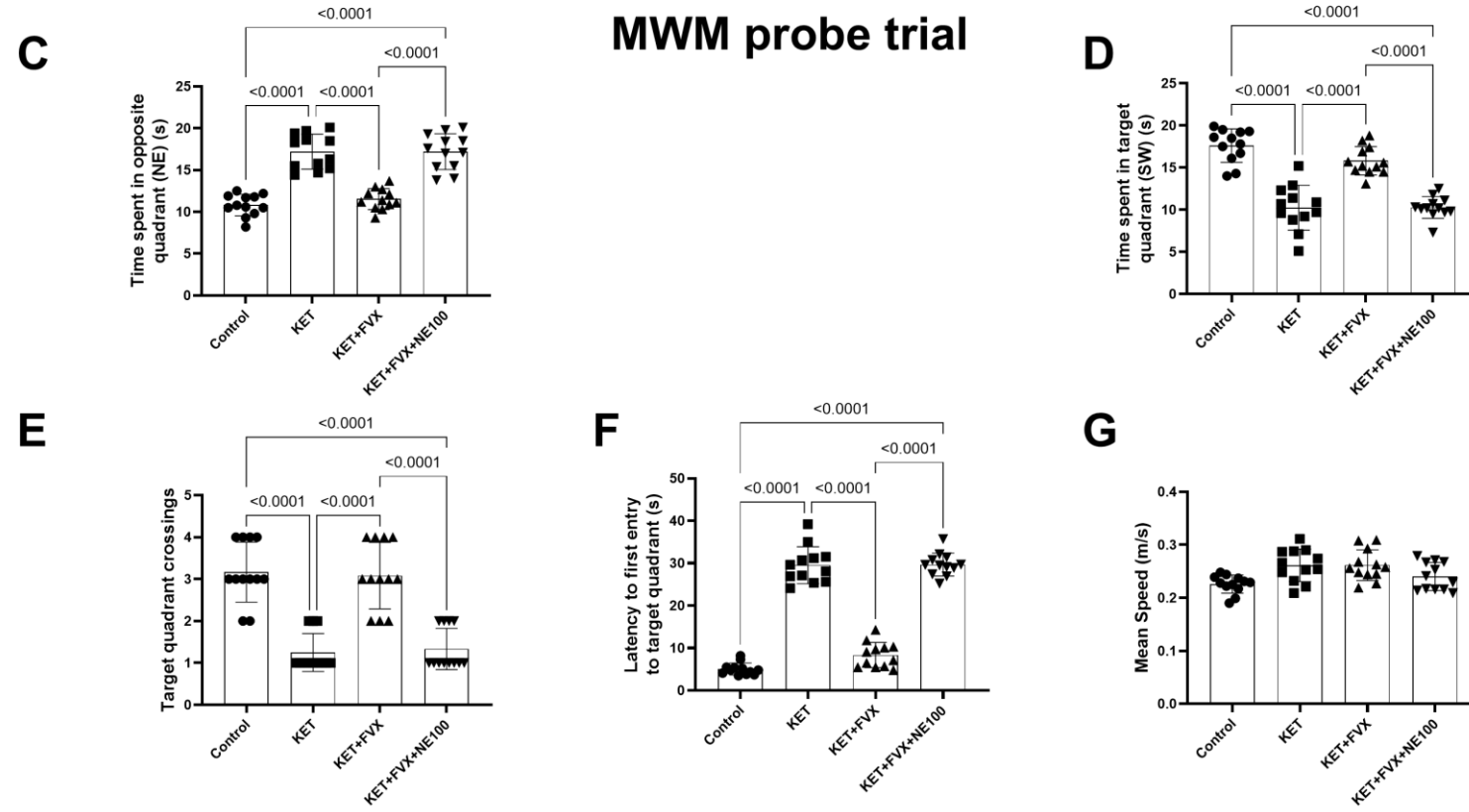

# Three-chamber Social Interaction Test

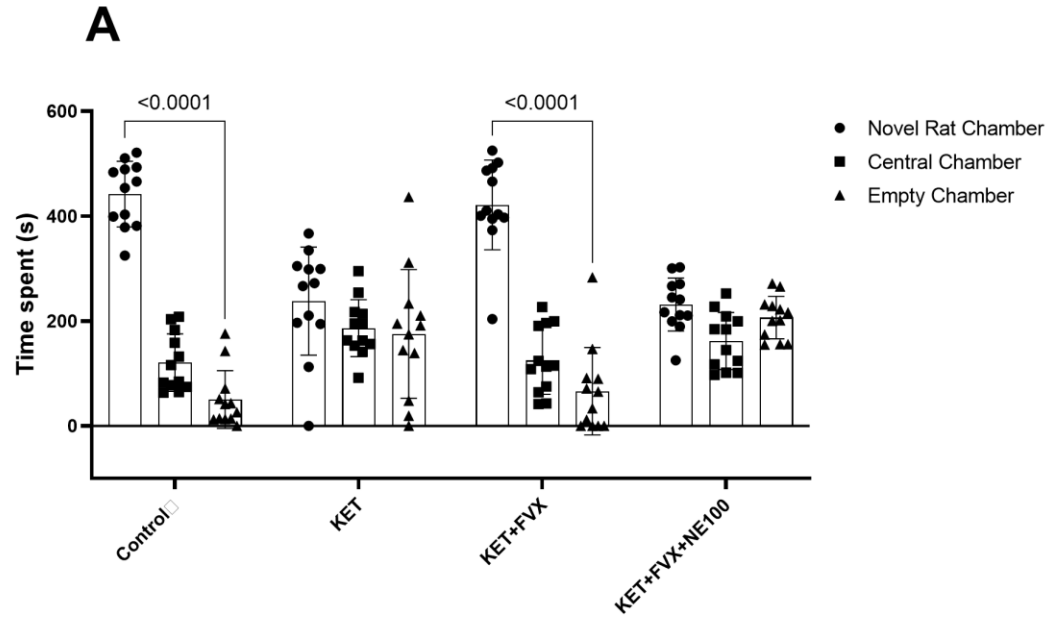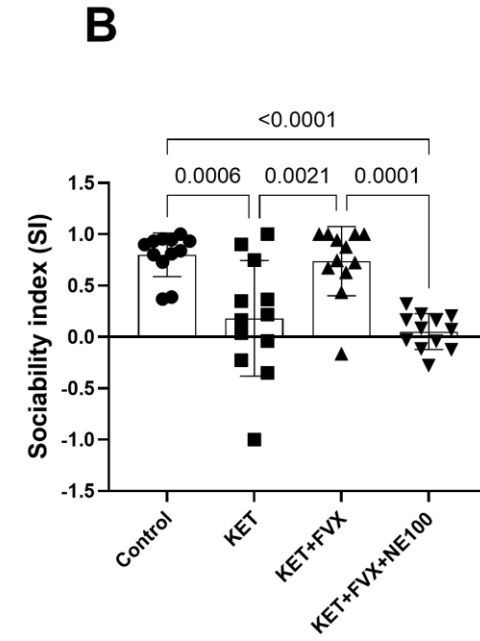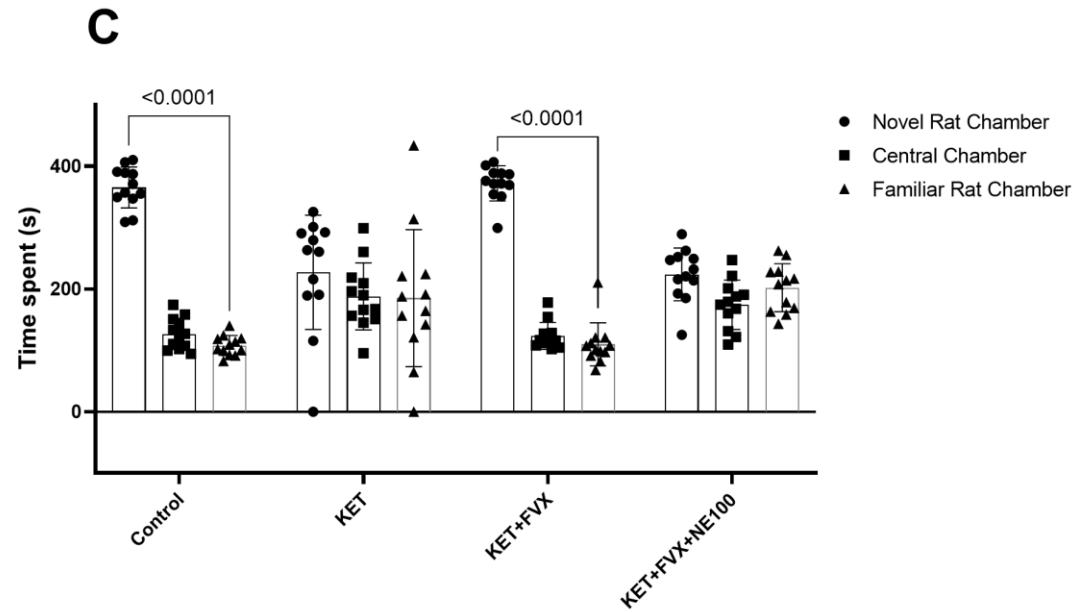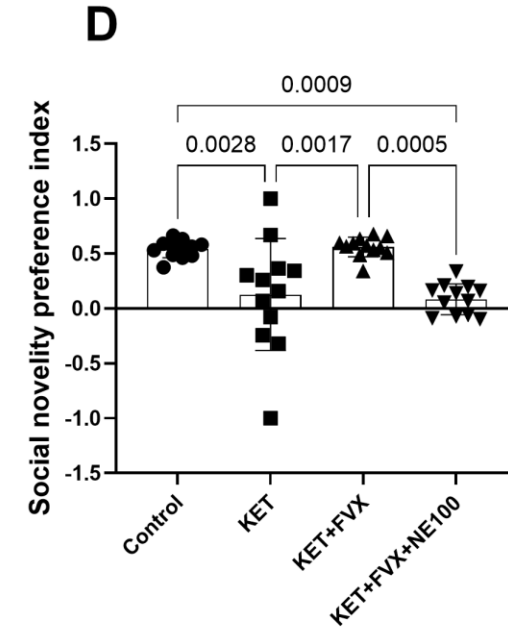

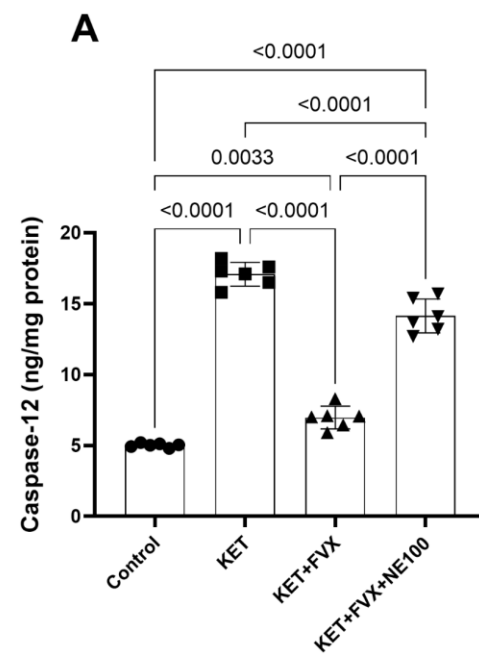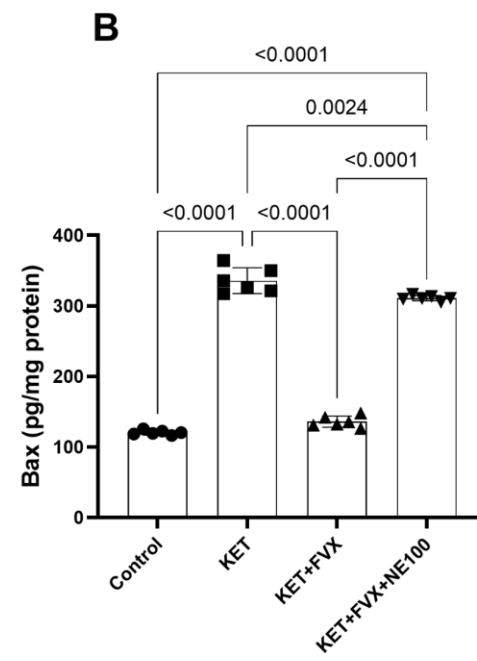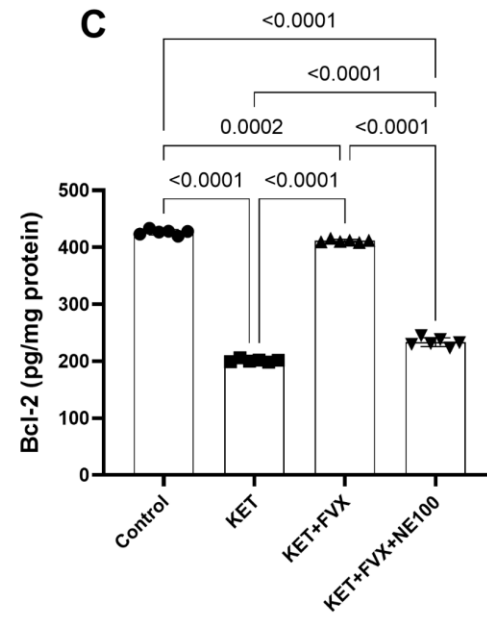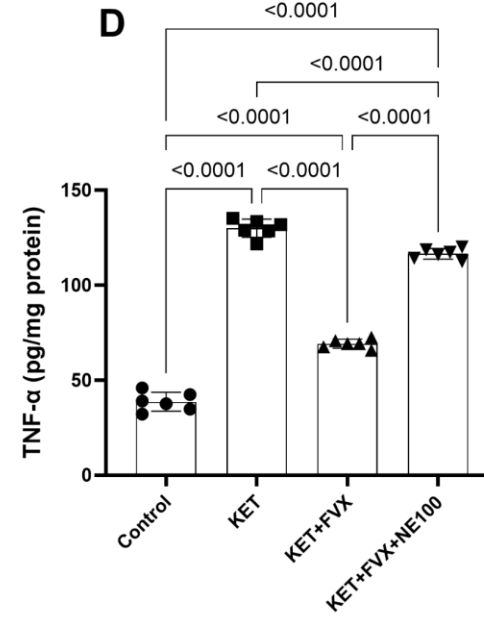

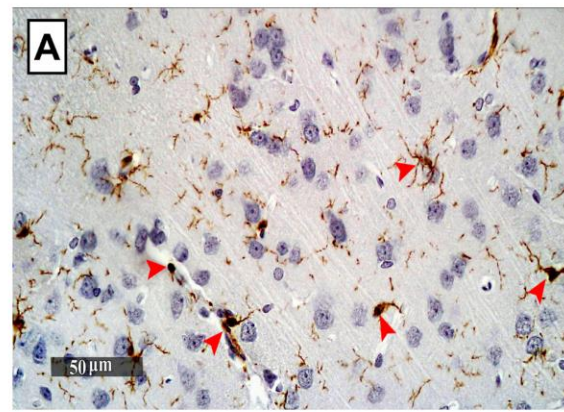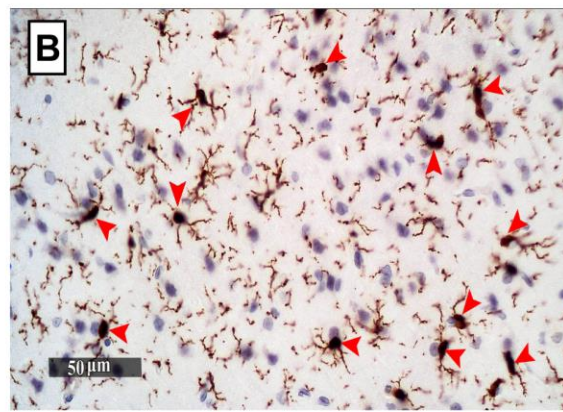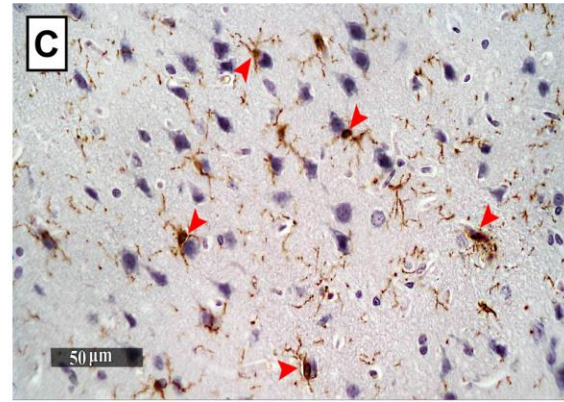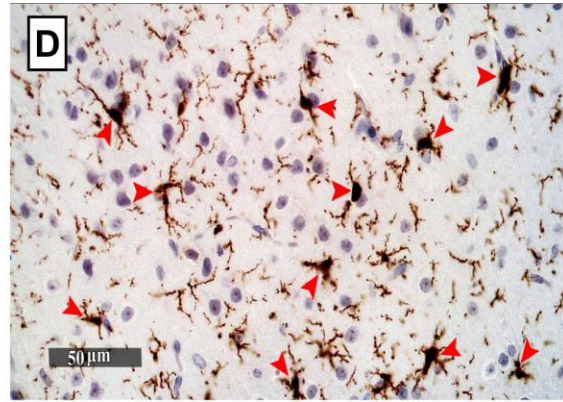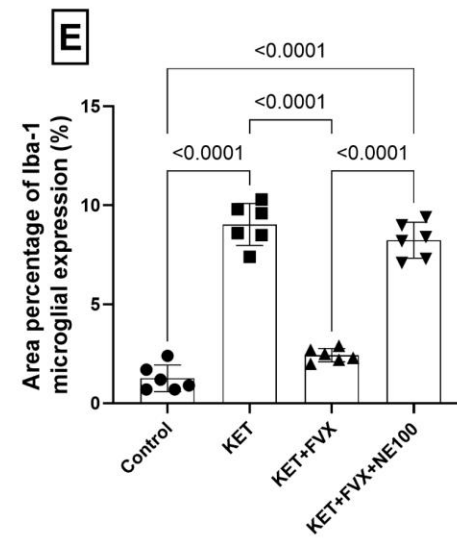

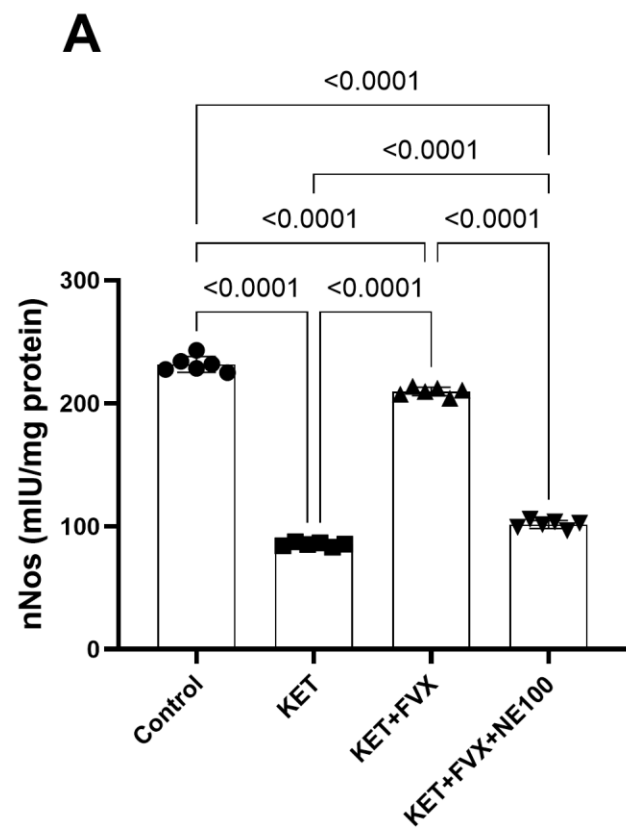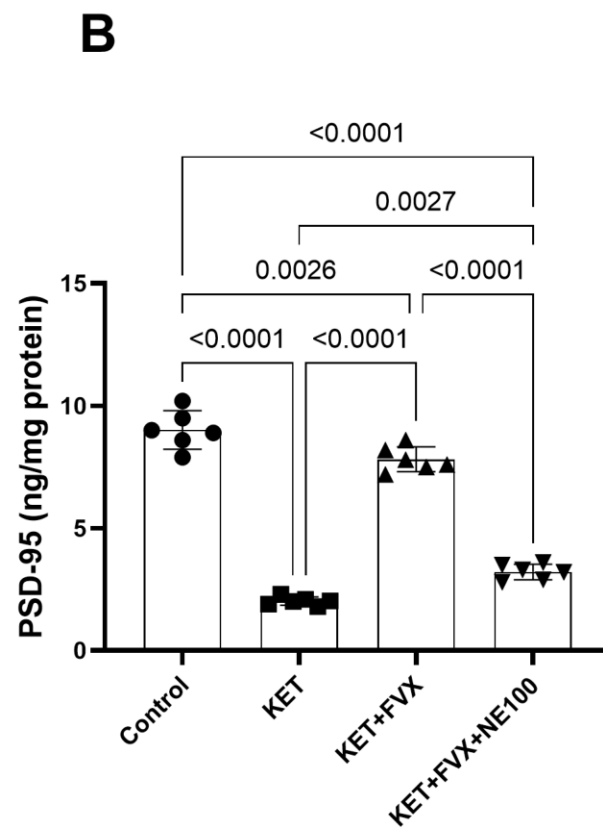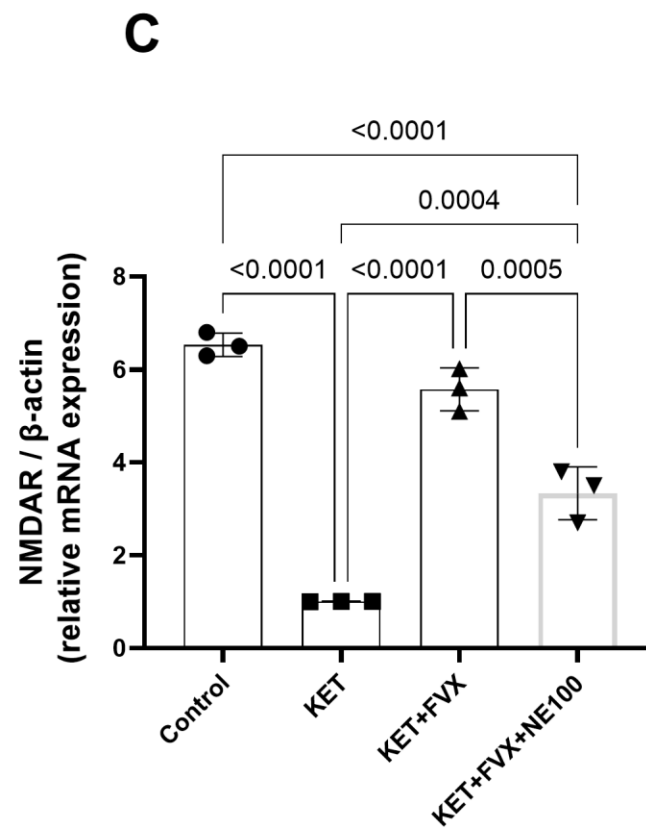

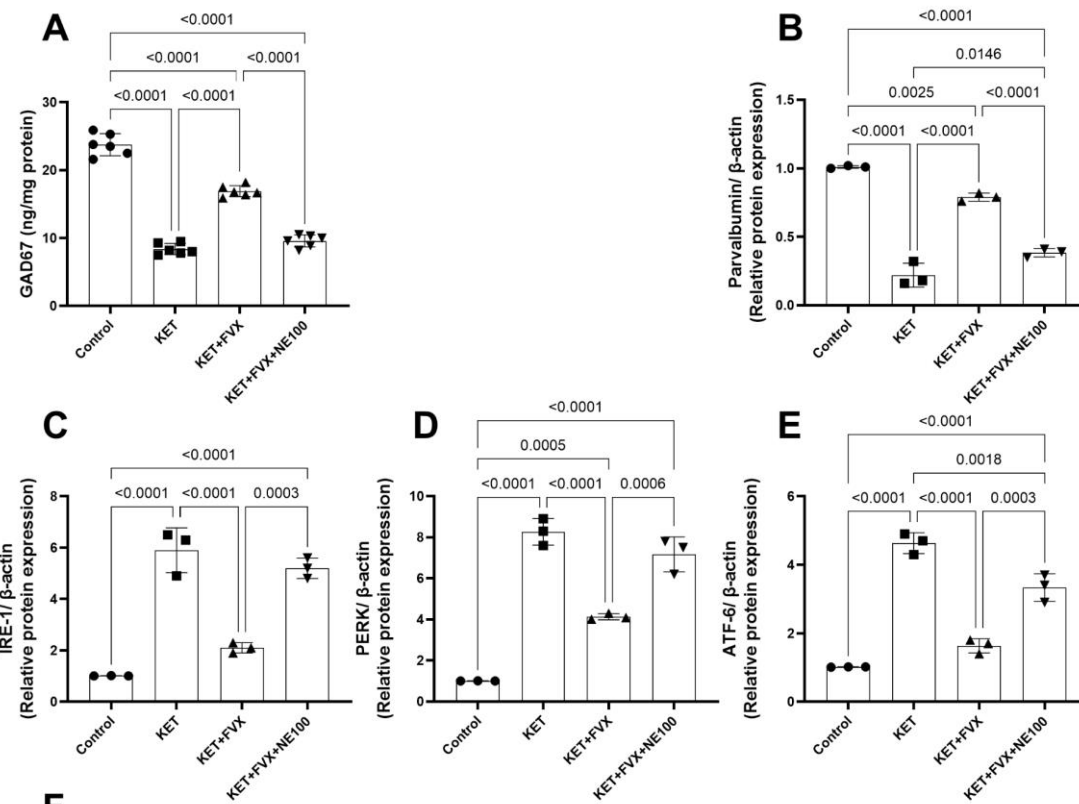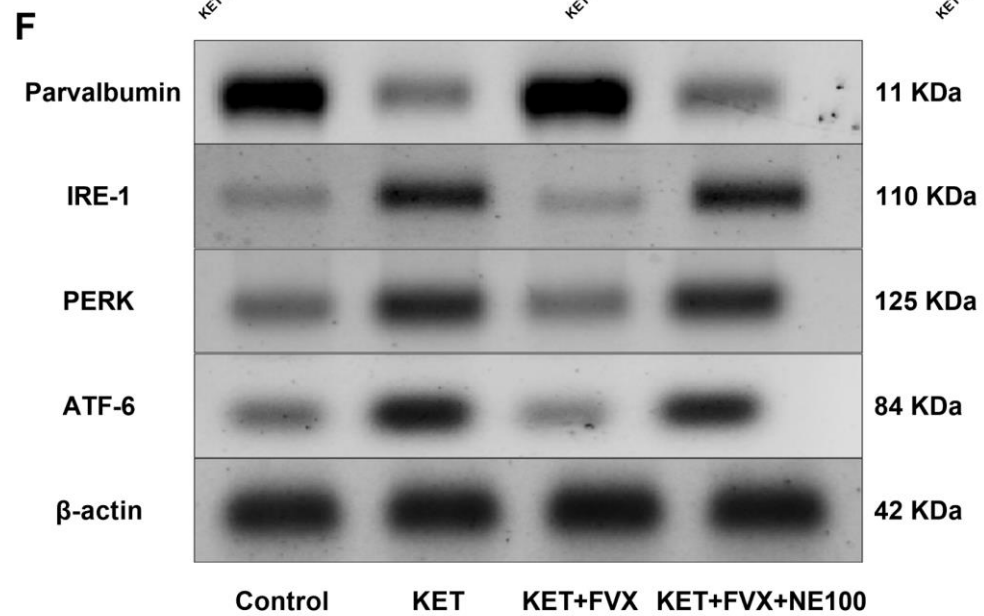

Supplement: Supplementary file 2 — Supplementary Material 2 [file 11481_2025_10231_MOESM2_ESM.pdf]
